# Molecular analysis of the *14-3-3* genes in *Panax ginseng* and their responses to heat stress

**DOI:** 10.7717/peerj.15331

**Published:** 2023-05-09

**Authors:** Qi Wang, Wenyue Peng, Junbo Rong, Mengyang Zhang, Wenhao Jia, Xiujuan Lei, Yingping Wang

**Affiliations:** College of Chinese Medicinal Materials, Jilin Agricultural University, Changchun, Jilin, China

**Keywords:** *14-3-3*, Abiotic stresses, Heat stress, qRT-PCR, Gene structure, Phylogenetic analysis, Ginseng

## Abstract

**Background:**

*Panax Ginseng* is a perennial and semi-shady herb with tremendous medicinal value. Due to its unique botanical characteristics, ginseng is vulnerable to various abiotic factors during its growth and development, especially in high temperatures. Proteins encoded by *14-3-3* genes form a highly conserved protein family that widely exists in eukaryotes. The *14-3-3* family regulates the vital movement of cells and plays an essential role in the response of plants to abiotic stresses, including high temperatures. Currently, there is no relevant research on the *14-3-3* genes of ginseng.

**Methods:**

The identification of the ginseng *14-3-3* gene family was mainly based on ginseng genomic data and Hidden Markov Models (HMM). We used bioinformatics-related databases and tools to analyze the gene structure, physicochemical properties, *cis*-acting elements, gene ontology (GO), phylogenetic tree, interacting proteins, and transcription factor regulatory networks. We analyzed the transcriptome data of different ginseng tissues to clarify the expression pattern of the *14-3-3* gene family in ginseng. The expression level and modes of *14-3-3* genes under heat stress were analyzed by quantitative real-time PCR (qRT-PCR) technology to determine the genes in the *14-3-3* gene family responding to high-temperature stress.

**Results:**

In this study, 42 *14-3-3* genes were identified from the ginseng genome and renamed *PgGF14-1* to *PgGF14-42*. Gene structure and evolutionary relationship research divided *PgGF14s* into epsilon (ε) and non-epsilon (non-ε) groups, mainly located in four evolutionary branches. The gene structure and motif remained highly consistent within a subgroup. The physicochemical properties and structure of the predicted *PgGF14* proteins conformed to the essential characteristics of *14-3-3* proteins. RNA-seq results indicated that the detected *PgGF14s* existed in different organs and tissues but differed in abundance; their expression was higher in roots, stems, leaves, and fruits but lower in seeds. The analysis of GO, *cis*-acting elements, interacting proteins, and regulatory networks of transcription factors indicated that *PgGF14s* might participate in physiological processes, such as response to stress, signal transduction, material synthesis-metabolism, and cell development. The qRT-PCR results indicated *PgGF14s* had multiple expression patterns under high-temperature stress with different change trends in several treatment times, and 38 of them had an apparent response to high-temperature stress. Furthermore, *PgGF14-5* was significantly upregulated, and *PgGF14-4* was significantly downregulated in all treatment times. This research lays a foundation for further study on the function of *14-3-3* genes and provides theoretical guidance for investigating abiotic stresses in ginseng.

## Introduction

During growth and development, plants are continuously affected by adverse factors, especially abiotic and biological stresses (Gong et al., 2020). In order to survive better, plants have derived a series of functional genes and regulatory mechanisms throughout the long evolutionary process, among which the *14-3-3* genes are crucial (Roberts, Salinas & Collinge, 2002). The *14-3-3* protein family is a group of highly conserved soluble acidic proteins widely distributed in various eukaryotic cells (Aitken, 2006). It was first discovered in bovine brain tissue and named according to the distribution of proteins in the diethylaminoethyl-cellulose column and mobility of starch gel electrophoresis (Tzivion, Shen & Zhu, 2001). Since encoded genes for the *14-3-3* proteins were identified in many stress-regulated genes, the family was first thought to be involved in abiotic stress responses (Roberts, Salinas & Collinge, 2002). Nowadays, plant 14-3-3 proteins are known to interact with many proteins and are also the primary messengers of signal transduction, which is necessary for abiotic stress responses (Liu, Zhang & Liu, 2016). In addition, with the improvement of proteomic technology and the maturity of the yeast hybridization system, studies on 14-3-3 proteins have gradually deepened and revealed their essential roles in plant signal transduction (Gampala et al., 2007), life process regulation (Mayfield, Paul & Ferl, 2012), resistance to stress (Paul et al., 2012), and material metabolism (Shin et al., 2011).

The *14-3-3* gene family was identified entirely in many plants, especially some model or economic plants, where they were cloned successfully for further analysis of their function and mechanism. Fifteen *14-3-3* genes have been identified in *Arabidopsis thaliana* and divided into ε/non-ε groups according to the core region of the protein, providing a reference for the subsequent study of *14-3-3* genes in other species (DeLille, Sehnke & Ferl, 2001). Moreover, 22 genes belonging to the *14-3-3* family have been identified in *Glycine max* (Wang et al., 2019), nine in *Citrus sinensis* (Lyu et al., 2021), eight in *Oryza sativa* (Yao et al., 2007), 25 in *Musa acuminat* (Li et al., 2016), seven in *Brachypodium distachyon* (Cao et al., 2016), 12 in *Solanum lycopersicum* (Xu & Shi, 2006), nine in *Phaseolus vulgaris* (Li et al., 2015), and 16 in *Mangifera indica* (Xia et al., 2022). The overexpression of the *AtGF14λ* gene in cotton allows the plant to stay green with higher photosynthesis under water deficit conditions (Yan et al., 2004). The salt overly sensitive (SOS) pathway regulates salt tolerance enhancement and maintains cellular sodium homeostasis. The interaction of the *AtGF14* gene with the structural domain of the *SOS2* gene affects enzyme activity and thus regulates plant salt tolerance in *A. thaliana* (Zhou et al., 2014). The heterologous expression of the maize *ZmGF14-6* gene in rice improves tolerance to drought stress while enhancing the response to pathogen invasion (Campo et al., 2012). Nitrate stress significantly increases the expression of the *Spinacia oleracea So14-3-3* gene in leaves and roots, and the interaction of *So14-3-3* with an H^+^-ATPase and a nitrate reductase (NR) was enhanced in transformed lines with higher enzymatic activity (Xu et al., 2016). Moreover, the 14-3-3 protein family is involved in hormone-mediated signal transduction and abiotic stress processes. Studies have demonstrated that 14-3-3 proteins can synergize with brassinolide (BA) (De Paepe et al., 2008), abscisic acid (ABA) (Takahashi, Kinoshita & Shimazaki, 2007; Chen et al., 2017), and gibberellin (GA) (Richards et al., 2001). Plasma membrane H^+^-ATPase also participates in the response of plants to various abiotic stresses, and 14-3-3 proteins could act as regulators of H^+^-ATPase activity. When H^+^-ATPase is affected by 14-3-3, the self-inhibition of the C-terminus of H^+^-ATPase is restricted, leading to an increased activity, and 14-3-3 proteins will preferably combine with H^+^-ATPase under stress conditions to react quickly (Bobik et al., 2010).

Ginseng (2*n* = 48) is a perennial herb with various medicinal and economic values that belongs to the Araliaceae family (Choi, 2008). Along with the extension of ginseng growth, more active ingredients and secondary metabolites accumulate, increasing their medicinal value (Lee et al., 2011). However, due to its long growth cycle (generally 5–6 years), ginseng is highly susceptible to various abiotic stresses that affect its yield and quality. Ginseng is a plant of high genuineness with an optimum growth temperature of 21–25 °C and sensitivity to temperature changes (Kim et al., 2019). The photosynthetic capacity of ginseng is closely related to light intensity and foliar temperature. As temperature increases, the respiration rate of ginseng increases, together with light, changing the plant’s photosynthetic rate. Among many unfavorable abiotic factors, high temperature is one of the main ones affecting the yield and quality of ginseng. During the hot planting season, the net photosynthesis rate, stomatal conductance, intercellular CO_2_ concentration, and transpiration rate of ginseng are disturbed due to the high-temperature injury (HTI) (Lee et al., 2012). After a week’s growth at 30 °C, the leaves will scorch, and the photosynthetic pigment structure will be destroyed, affecting the photosynthesis and growth of ginseng and even leading to the plant’s death (Lee, Lee & Ahn, 2010). Breeding stress-tolerant varieties is one of the main ways to reduce the adverse effects of abiotic stress, and resistance genes are essential for this work. Therefore, it is necessary to mine related genes (Li et al., 2022). The development of molecular biology techniques and the refinement of genomes have provided ideas and methods for studying stress resistance in species. The ginseng genome is well-developed to facilitate the study of functional genes. *14-3-3* genes are critical in plant responses to abiotic stresses, but the ginseng *14-3-3* gene family has not been studied so far. In this study, we identified the *14-3-3* family of ginseng based on the whole genome data for the first time and predicted the structure, function, evolutionary relationships, and interactions of *14-3-3* genes from multiple perspectives and levels. RNA-seq data were analyzed to get the gene expression pattern in different tissues, and a qRT-PCR experiment was conducted to explore the response of *14-3-3* genes to heat stress. The study lays the foundation for researching abiotic stresses in ginseng and provides a reference for mining essential functional genes.

## Materials and Methods

### Gene family identification and physicochemical property analysis

Genome data for ginseng were generated from the leaves of a four-year-old Korean cultivar ‘Chunpoong,’ and three individuals were used to reduce heterogeneity. The reads were obtained by the Illumina platform (HiSeq2000 and MiSeq), and the sequences were assembled based on paired-end and mate-paired raw data. Genome and annotation files were available from the Ginseng Genome Database (http://ginsengdb.snu.ac.kr/; Jayakodi et al., 2018). The HMM of *14-3-3* genes was retrieved and downloaded from the Pfam database (Bateman et al., 2004) with access number PF00244, and the E-value threshold was 10^−5^. The conserved structural domains of the genes were further identified by the SMART (Letunic, Doerks & Bork, 2015) database (http://smart.embl.de/) and NCBI-Conserved Domain Database (NCBI-CDD), and genes lacking the target structural domains were excluded. The ExPASy program (http://www.ExPASy.org/tools; Rueda et al., 2015) was used to predict the features of 14-3-3 proteins, including molecular weight, isoelectric point, stability, and hydrophilicity. Potential signal peptide cleavage sites in proteins were predicted by SiginalP-5.0 (https://services.healthtech.dtu.dk/service.php?SignalP-5.0). The prediction of protein transmembrane helix structure was implemented by TMHMM-2.0 (https://services.healthtech.dtu.dk/service.php?TMHMM-2.0). SOPMA (https://npsa-prabi.ibcp.fr/cgi-bin/npsa_automat.pl?page=npsa%20_sopma.html) and SWISS-MODEL (https://swissmodel.expasy.org) were used to predict protein secondary and tertiary structures, respectively (Biasini et al., 2014). Finally, the subcellular localization prediction tools WoLF PSORT (https://wolfpsort.hgc.jp) and CELLO (http://cello.life.nctu.edu.tw/) were used to preliminarily determine the location of the family protein or its gene expression product in the cell (Yu, Lin & Hwang, 2004; Horton et al., 2007).

### Gene structure and motif analysis

Based on the genome annotation information, the Gff3 file of the *14-3-3* gene family was obtained to analyze the gene structure. The motif was analyzed using the MEME online tool (Bailey et al., 2009; https://meme-suite.org/meme/). The number of motifs was set to ten and the motif width option to 6–50, and the remaining parameters were left on default. Based on TBtools software (Chen et al., 2020), the gene structure and MEME output information were visualized.

### *Cis*-acting elements and GO analysis

The 2,000-bp sequence upstream of the gene coding sequence (CDS) was extracted from the ginseng genomic data by annotation files, and the upstream sequence of the ginseng *14-3-3* genes was extracted from the collection based on the gene ID and uploaded to the PlantCARE database (http://bioinformatics.psb.ugent.be/webtools/plantcare/html/) to predict the type and function of the *cis*-acting elements (Lescot et al., 2002). To further explore the function of *14-3-3* genes, the protein sequences were uploaded to the eggNOG database (http://eggnog-mapper.embl.de/) to obtain GO terms and corresponding descriptive information of the target genes (Cantalapiedra et al., 2021). Each GO term is at a node; the distance from that node to the root node is the level number. Based on the TBtools (GO Level Count) and GO background files (http://geneontology.org/docs/download-ontology/#go_basic), the predicted results were filtered and analyzed at level 2. Results for GO analysis were then visualized according to the number of genes involved in the biological process (BP), cellular component (CC), and molecular function (MF) in the *14-3-3* gene family.

### Evolutionary tree analysis of the ginseng *14-3-3* gene family

*Daucus carota*, *A. thaliana*, *Gossypium hirsutum*, and *O. sativa 14-3-3* genes were selected to construct an evolutionary tree with the *P. ginseng 14-3-3* gene family. *D. carrot 14-3-3* genes were retrieved from the UniProt (Apweiler, 2008) protein database (https://www.uniprot.org). Following gene ID, the *A. thaliana 14-3-3* genes were obtained from the Arabidopsis Information Resource (https://www.arabidopsis.org/). The *G. hirsutum 14-3-3* genes were downloaded from the Cottongen database (https://www.cottongen.org/), and the *O. sativa 14-3-3* genes were obtained from the NCBI by accession number. Multiple *14-3-3* protein sequences were aligned by Multiple Sequence Comparison by Log-Expectation (MUSCLE) within 1,000 bootstrap replications (Manuel, 2013). The comparison results were further cut and processed by trimAl, and the evolutionary tree was constructed by IQ-tree (v1.6.12) to filter the most suitable amino acid replacement model. The output tree file was uploaded to the ITOL tool (https://itol.embl.de/itol.cgi) to annotate and optimize the tree diagram (Letunic & Bork, 2021).

### Interaction protein and transcription factor regulatory network analysis

The String database (https://cn.string-db.org/) was used to screen for proteins with potential interactions with ginseng *14-3-3* genes (Szklarczyk et al., 2021). Protein sequences were mapped to the most homologous genes, the interaction score was 0.7, and the max number beyond the level 1 shell was no more than 20 interactors. Based on the genome and annotation information, the 600 bp sequence upstream of *14-3-3* genes was extracted by TBtools (Chen et al., 2020). The regulation prediction module of the Plant Transcriptional Regulatory Map database (http://plantregmap.gao-lab.org/) was used to predict transcription factors that have potential regulatory interactions with the input sequences (Tian et al., 2020). The analysis results were downloaded locally, then the protein interaction network and transcription factor regulatory network were constructed by Cytoscape (Shannon et al., 2003).

### Analysis of *14-3-3* family expression among different tissue of ginseng

In order to clarify whether the *14-3-3* gene family has tissue or organ expression differences in ginseng, a transcriptomic data analysis was performed (Wang et al., 2015). Raw data for different tissues of ginseng were downloaded from NCBI with the accession number PRJNA302556. Raw data quality was checked with FastQC, and low-quality reads were removed with Trimmomatic. Reads were matched to the corresponding positions in the ginseng reference genome through Tophat v.2.0.10, and the transcriptome was assembled using Cufflinks (Trapnell et al., 2012). Transcripts were quantified using Features Counts, and a matrix was made to measure gene expressions as normalized transcript per million (TPM) values. Next, the TPM values were transformed and normalized in log_2_ (TPM+1), and a heat map of the expression of *14-3-3* genes in different tissues was generated with the euclidean and complete parameters for clustering.

### RNA extraction and expression analysis of ginseng *14-3-3* genes under heat stress

The relative expression of target genes was probed by qRT-PCR to determine whether ginseng *14-3-3* genes responded to high temperature. Their expression was analyzed under different processing times. Ginseng tissue-cultured ‘Fu Xing 1’ seedlings in their leaf-expansion stage (15–20 d) were subjected to high-temperature stress at 35 °C for 0, 12, 24, and 36 h, and three biological replicates were used. The petioles and leaves of the seedlings were quickly placed in liquid nitrogen at the corresponding stress points and stored at −80 °C. Mixed RNA samples from petioles and leaves were extracted using RNAprep Pure Plant Kit (DP441; TIANGEN, Beijing, China) and quantified by NanoPhotometer N50 (Implen, Munich, Germany), and the integrity was checked on a 1% agarose gel. The mRNA was reverse transcribed into cDNA using the TransScript^®^ II One-Step RT-PCR SuperMix (AH411; TransGen, Beijing, China). Specific primers were designed and synthesized for quantitative gene analysis using the *β-Actin* gene (NCBI: No. AY907207) as a reference; the primer sequences are displayed in Table S1. The gene analysis was done by a two-step method based on the TransStart^®^ Green qPCR SuperMix (AQ101-01; TransGen, Beijing, China) in LightCycler 96 (Roche, Basel, Switzerland) with a reaction system of pre-denaturation at 95 °C for 30 s, and 40 thermocycles of 94 °C for 5 s and 60 °C for 30 s. The obtained melting curve helped determine the specificity of primers and genes. Two sets of technical replicates were set up for each sample. Experimental data were processed and analyzed using the 2^−∆∆CT^ method (Livak & Schmittgen, 2001) by Cq value. The expression of the target gene in the control group (0 h) was defined as 1, and thus calculate the fold change of gene expression after heat stress. Statistically significant differences were analyzed and are indicated with asterisks with *p < 0.05 and **p < 0.01 based on independent Student’s T-tests at the top of the columns, with error bars representing the SD for three biologic replicates and two technical replicates.

## Results

### *14-3-3* gene family identification and physicochemical property analysis

According to the HMM model combined with SMART and NCBI-CDD co-identification, 42 *14-3-3* genes were identified in ginseng with the conserved structural domain of *14-3-3* proteins and named *PgGF14-1* to *PgGF14-42* (Fig. S1). Their basic characteristics, physicochemical properties, and subcellular localization were analyzed with the support of bioinformatics (Tables 1 and S2). The full lengths ranged from 402 bp for *PgGF14-1* to 15,261 bp for *PgGF14-42*. The CDS and corresponding amino acids ranged from 291 bp and 96 aa for *PgGF14-12* to 2,052 bp and 683 aa for *PgGF14-42*. However, the CDS length of most genes was below 1,000 bp. The proteins’ molecular weight (MW) ranged from 11.27 kDa for *PgGF14-12* to 76.98 kDa for *PgGF14-42*. Except for *PgGF14-38*, *41*, and *42*, which had larger molecular weights, the rest of the PgGF14 proteins ranged from 11.27 to 31.35 kDa. All proteins’ isoelectric points (pI) were lower than 7, except for *PgGF14-5* and *PgGF14-15*, indicating that PgGF14s are mainly acidic. Most proteins had an instability index (II) higher than 40, and their GRAVY values were all negative, indicating that PgGF14s are mostly unstable and hydrophilic. The subcellular localization results obtained from WoLF PSORT and CELLO were consistent, indicating that most PgGF14s are cytoplasmic and nuclear, *PgGF14-5* and *PgGF14-15* are mitochondrial, and only *PgGF14-27* is located in the plasma membrane, suggesting a diversity of action sites and functions (Table S2). In addition, none of the proteins had signal peptide shear sites and transmembrane structures, except *PgGF14-3* for the latter (Table S2). The secondary structures (Fig. S2) of PgGF14s indicated that most proteins were α-helices, with less β-folding and irregular coiling. Their tertiary structure (Fig. S3) demonstrated that the proteins primarily existed as dimers, and the monomers were mainly composed of multiple reverse parallel α-helices. The protein conformation mostly displayed a cup-like structure with grooves, which may be crucial in protein phosphorylation and target-protein interactions.

**Table 1:** Information about *PgGF14s* in the genome and essential characteristics.

Gene name	Gene ID	Gene locus	Length (bp)	CDS	Protein (AA)	Molecular weight (kDa)
*PgGF14-1*	Pg_S0049.6	Pg_scaffold0049:860907-861308	402	402	133	15.17
*PgGF14-2*	Pg_S0382.5	Pg_scaffold0382:635222-635659	438	318	105	12.20
*PgGF14-3*	Pg_S0778.7	Pg_scaffold0778:803749-804294	546	546	181	20.89
*PgGF14-4*	Pg_S2850.2	Pg_scaffold2850:85942-86723	782	327	108	12.24
*PgGF14-5*	Pg_S1006.2	Pg_scaffold1006:118529-119709	1,181	333	110	12.43
*PgGF14-6*	Pg_S0105.3	Pg_scaffold0105:381088-382593	1,506	516	171	18.36
*PgGF14-7*	Pg_S1029.36	Pg_scaffold1029:525401-527156	1,756	771	256	28.84
*PgGF14-8*	Pg_S8664.5	Pg_scaffold8664:25524-27288	1,765	771	256	28.83
*PgGF14-9*	Pg_S2461.5	Pg_scaffold2461:117774-119595	1,822	780	259	29.08
*PgGF14-10*	Pg_S0604.6	Pg_scaffold0604:895405-897276	1,872	780	259	29.09
*PgGF14-11*	Pg_S0207.2	Pg_scaffold0207:141328-143317	1,990	792	263	29.66
*PgGF14-12*	Pg_S2535.1	Pg_scaffold2535:340219-342214	1,996	291	96	11.27
*PgGF14-13*	Pg_S0146.2	Pg_scaffold0146:197765-199786	2,022	312	103	11.71
*PgGF14-14*	Pg_S2827.2	Pg_scaffold2827:196533-198567	2,035	534	177	19.93
*PgGF14-15*	Pg_S4893.15	Pg_scaffold4893:124689-126842	2,154	516	171	18.98
*PgGF14-16*	Pg_S2001.1	Pg_scaffold2001:58331-60520	2,190	471	156	18.10
*PgGF14-17*	Pg_S2151.5	Pg_scaffold2151:416939-419222	2,284	402	133	15.13
*PgGF14-18*	Pg_S2020.18	Pg_scaffold2020:174926-177250	2,325	783	260	29.68
*PgGF14-19*	Pg_S1750.41	Pg_scaffold1750:490928-493404	2,477	783	260	29.39
*PgGF14-20*	Pg_S2708.12	Pg_scaffold2708:118724-121264	2,541	783	260	29.25
*PgGF14-21*	Pg_S2390.8	Pg_scaffold2390:333170-335711	2,542	783	260	29.25
*PgGF14-22*	Pg_S2162.22	Pg_scaffold2162:232004-234703	2,700	783	260	29.68
*PgGF14-23*	Pg_S2402.1	Pg_scaffold2402:319884-322690	2,807	522	173	19.02
*PgGF14-24*	Pg_S1693.13	Pg_scaffold1693:212202-215071	2,870	537	178	20.04
*PgGF14-25*	Pg_S1497.31	Pg_scaffold1497:372682-375630	2,949	783	260	29.39
*PgGF14-26*	Pg_S8682.2	Pg_scaffold8682:14105-17100	2,996	780	259	29.71
*PgGF14-27*	Pg_S0909.4	Pg_scaffold0909:154974-158087	3,114	1,260	419	28.67
*PgGF14-28*	Pg_S1794.8	Pg_scaffold1794:277410-280735	3,326	759	252	28.48
*PgGF14-29*	Pg_S0061.54	Pg_scaffold0061:1331727-1336091	4,365	816	271	31.07
*PgGF14-30*	Pg_S5218.7	Pg_scaffold5218:122124-126800	4,677	789	262	25.83
*PgGF14-31*	Pg_S3744.4	Pg_scaffold3744:36630-41497	4,868	786	261	29.62
*PgGF14-32*	Pg_S1055.45	Pg_scaffold1055:68797-73670	4,874	417	138	15.76
*PgGF14-33*	Pg_S2588.15	Pg_scaffold2588:220870-225798	4,929	783	260	29.55
*PgGF14-34*	Pg_S5121.3	Pg_scaffold5121:50343-55482	5,140	789	262	29.57
*PgGF14-35*	Pg_S3628.13	Pg_scaffold3628:238426-243899	5,474	783	260	29.51
*PgGF14-36*	Pg_S6072.5	Pg_scaffold6072:50899-56478	5,580	753	250	28.17
*PgGF14-37*	Pg_S4884.1	Pg_scaffold4884:47869-54409	6,541	831	276	30.98
*PgGF14-38*	Pg_S0399.53	Pg_scaffold0399:798712-805592	6,881	1,311	436	49.01
*PgGF14-39*	Pg_S4151.1	Pg_scaffold4151:27680-34883	7,204	846	281	31.35
*PgGF14-40*	Pg_S3563.1	Pg_scaffold3563:177097-189293	12,197	759	252	28.42
*PgGF14-41*	Pg_S1590.20	Pg_scaffold1590:201614-216669	15,056	1,737	578	64.72
*PgGF14-42*	Pg_S2148.5	Pg_scaffold2148:42550-57810	15,261	2,052	683	76.98

### Gene structure and motif analysis

**Figure 1:**
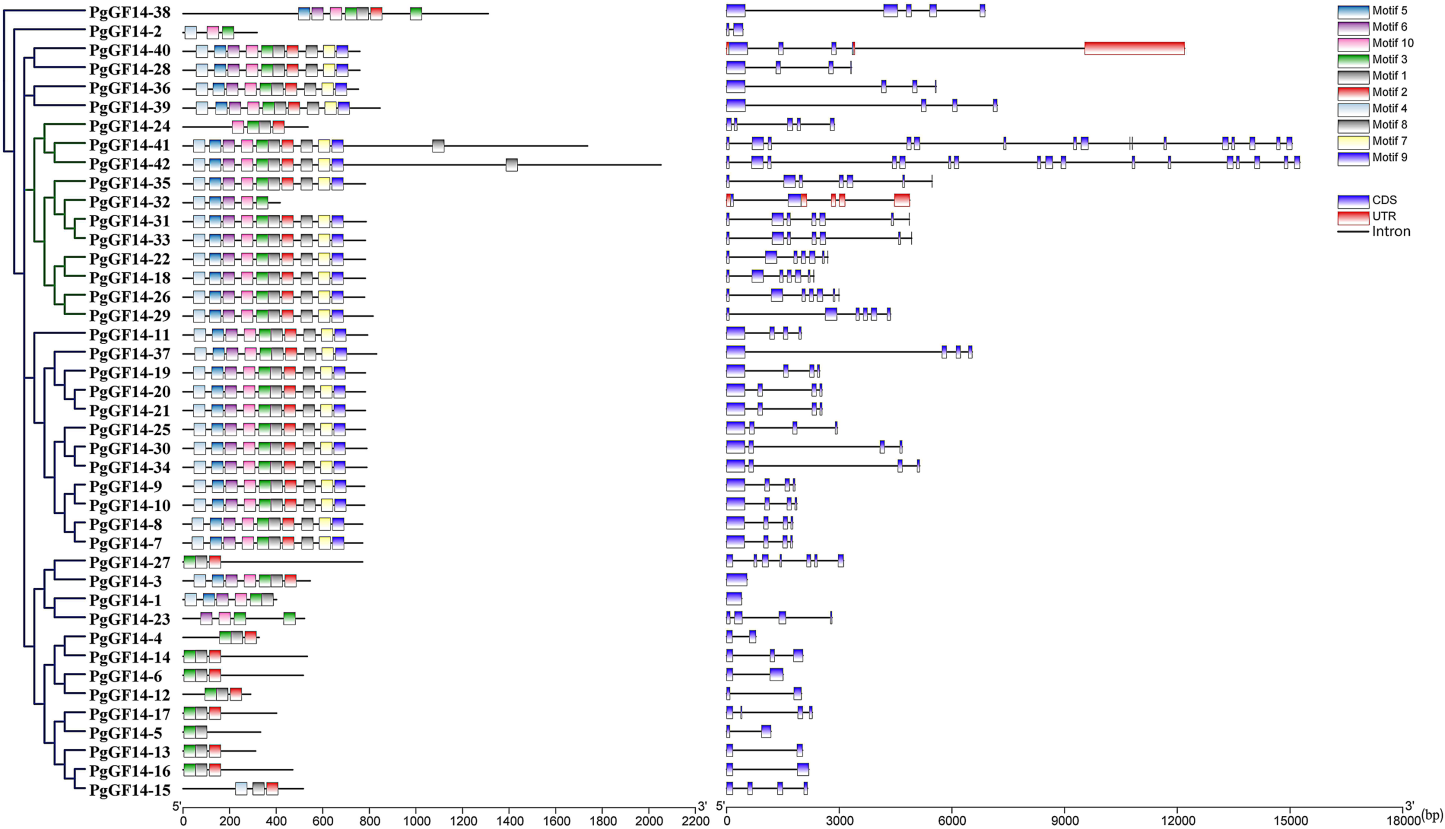
Conserved motifs and structure analysis for *PgGF14s*.

The gene intron-exon structure is unique to eukaryotes. It has been linked to the evolutionary process of gene families. The variability of intron-exon structures among different genes of a family also predicts the diversity of gene expansion and species evolution (Xu et al., 2019). The protein sequences of 42 genes were aligned using the MUSCLE program of MEGA 7.0, and the evolutionary tree of *PgGF14s* was constructed by the maximum likelihood (ML) method (Fig. 1). The alignment results indicated that PgGF14s displayed a high sequence similarity and strong conservativeness between them, but their C-terminus was variable (Fig. S4). Their structure (Fig. 1) also displayed significant differences, and most genes had two to seven exons. Based on their intron-exon structure, the genes were divided into two major classes, ε and non-ε groups. The ε class genes were located in the same major evolutionary branch. The number of exons of non-ε genes was mainly 2–4, except *PgGF14-1* with only one exon, *PgGF14-27* with six exons, and *PgGF14-38* with five exons. The number of exons of ε genes mainly was 5–7, except *PgGF14-41* with 16 exons and *PgGF14-42* with 17 exons. Genes displaying a similar structure can have variable intron lengths. The analysis of gene motifs (Fig. 1) indicated that *PgGF14s* were highly conserved, especially their motifs within the same evolutionary branch with good consistency. In contrast, the number and types of motifs in different evolutionary branches differed considerably. The motif sequences are provided in Fig. S5. Motifs 1, 2, and 3 were the most widely distributed in the gene family. Twenty-five *PgGF14s* contained all motifs, and *PgGF14-41*, *PgGF14-42* from the ε group had two motif 1. Seven genes included only motifs 1, 2, and 3. Other genes varied widely in the number and type of motifs. *PgGF14-5* had only motif 3 and motif 8, and *PgGF14-15* had motifs 2, 4, and 8. Except for *PgGF14-24* and *PgGF14-32*, nine genes of class ε had motifs 1 to 10. Although another 16 genes in the non-ε group were highly consistent with the motifs of these nine class ε genes, their structures were very different, possibly related to gene duplication events.

### *14-3-3* gene family *cis*-acting elements and GO analysis

**Figure 2:**
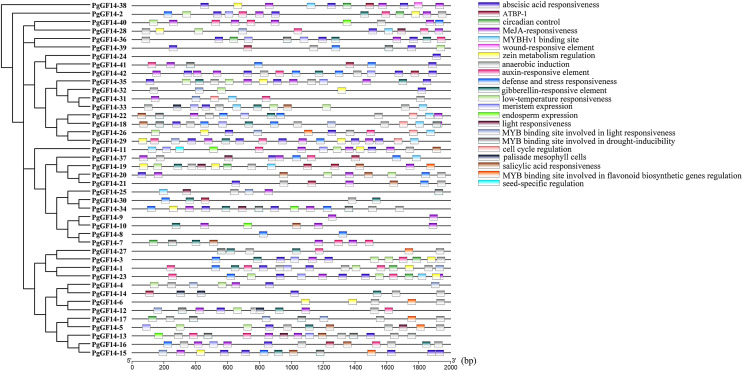
Analysis of upstream *cis*-acting elements for *PgGF14s*. The different colour blocks symbolize the different *cis*-acting element types, and the legend on the right illustrates the possible biological functions of the elements.

Gene expression is the result of multi-level and multi-factor regulation. The *cis*-acting element is a segment of the DNA sequence that affects gene expression and is essential in site binding. The results and details of the PlantCARE analysis about *PgGF14s cis*-element types and roles are displayed in Table S3. Twenty-two kinds of *cis*-acting elements were predicted in the upstream sequences of *PgGF14s*, mainly including hormone-responsive, light-responsive, stress-responsive, and growth and development regulatory elements (Fig. 2). Five hormone elements responding to ABA, methyl jasmonate (MeJA), auxin (IAA), GA, and salicylic acid (SA) were more widely distributed in the upstream regulatory regions of *PgGF14s* with corresponding ABRR, TGACG, TGA-motif, P-box, and TCA-element, suggesting that multiple hormones may induce the expression of the ginseng *14-3-3* genes. In addition, 32 genes had the anaerobic induction element ARE, and 19 *PgGF14s* had TC-rich repeats and binding sites for defense and stress response elements. Seventeen *PgGF14s* had LTR-based low-temperature response elements upstream of the gene. Upstream sequences of ten genes also contained AT-rich DNA binding protein (ATBP-1), the binding site for AT-rich elements. Some genes that contained upstream binding sites for MYB and MYBHv1 were involved in light responses, drought-inducibility, and flavonoid biosynthetic gene regulation. Furthermore, circadian control, endosperm expression, meristem expression, and zein metabolism regulation elements were also found upstream of the *14-3-3* genes. Two elements were specific to certain genes: a seed-specific regulatory element was found upstream of *PgGF14-11*, and a wound-responsive element upstream of *PgGF14-38*. The abovementioned results suggest that the *14-3-3* gene family may be involved in physiological processes, such as growth and development regulation, stress response, substance metabolism, and hormone response in ginseng.

**Figure 3:**
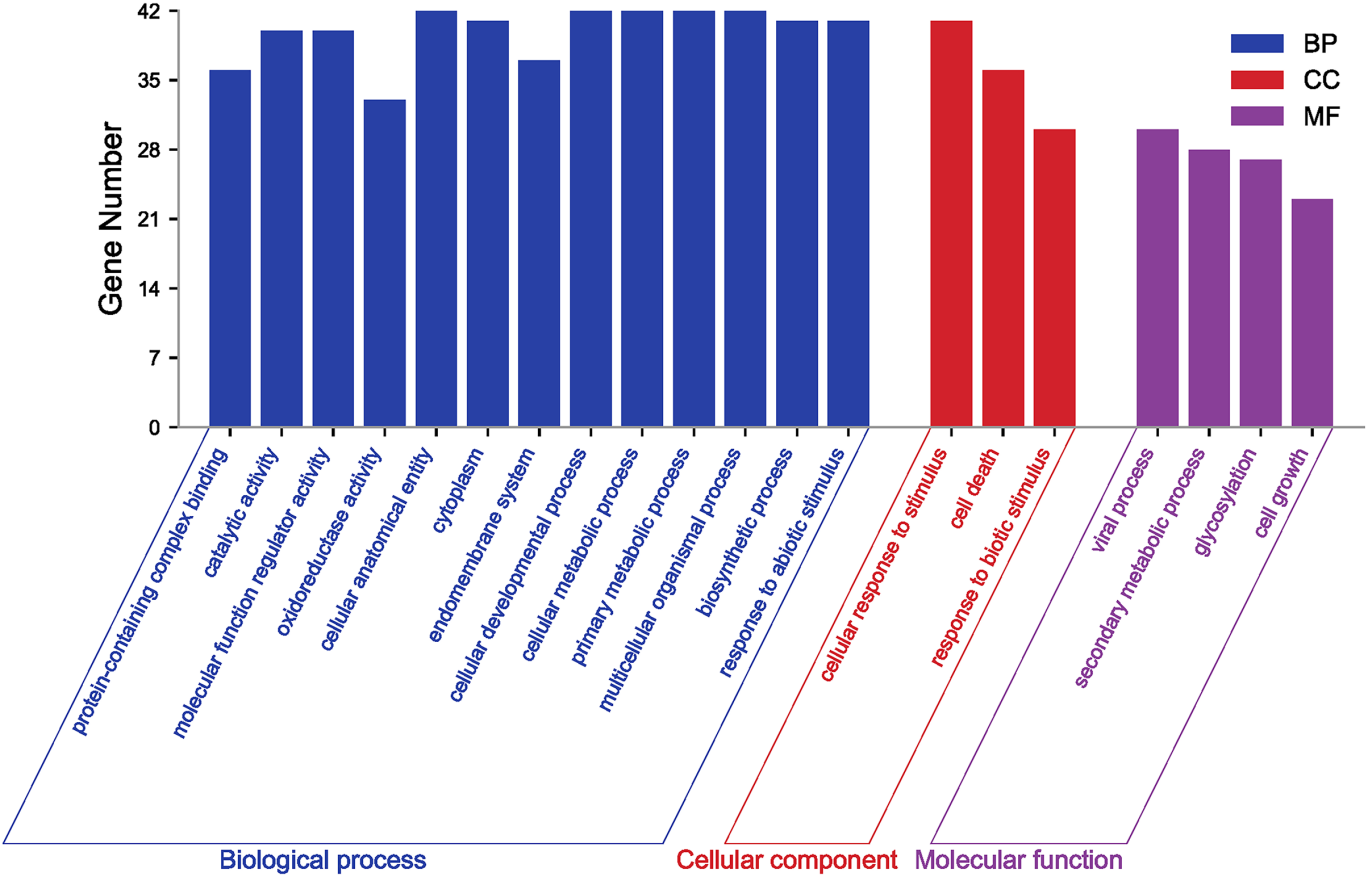
GO enrichment analysis for *PgGF14s* in ginseng.

GO annotation and enrichment analysis of genes effectively clarify genes’ biological functions and the processes they are involved in. In order to better analyze the results of GO, it is usually necessary to filter all terms. Here, GO terms for *PgGF14s* were obtained by eggNOG and further enriched by TBtools at level 2 for analysis. Details of GO annotations and analysis results are displayed in Table S4. *PgGF14s* were enriched in biological process, cellular component, and molecular function process terms (Fig. 3). All members were enriched in cellular developmental process (GO:0048869), cellular metabolic process (GO:0044237), primary metabolic process (GO:0044238), multicellular organismal process (GO:0032501), and cellular and anatomical entity (GO:0110165). Forty *PgGF14s* were related to responses to abiotic stimuli (GO:0009628) and cellular response to stimuli (GO:0051716), and 30 PgGF14s were found to respond to biotic stimuli (GO:0009607). These findings reflected the involvement of *PgGF14s* in biological processes in response to stresses from a gene function level. *PgGF14s* were also associated with biosynthesis and substance metabolism, such as biosynthetic processes (GO:0009058), primary metabolic processes (GO:0044238), secondary metabolic processes (GO:0019748), and the life activities of cells (GO:0016049, GO:0008219). From the perspective of molecular function, *PgGF14s* were mainly associated with the catalysis of specific reactions and protein-containing. The enrichment results of the cellular component also predicted a wide distribution of *14-3-3* genes in cells. Overall, the results of the GO analysis indicated that ginseng *14-3-3* genes were involved in various life activities, which is consistent with the functional diversity of the *14-3-3* gene family.

### Evolutionary tree analysis of the ginseng *14-3-3* gene family

**Figure 4:**
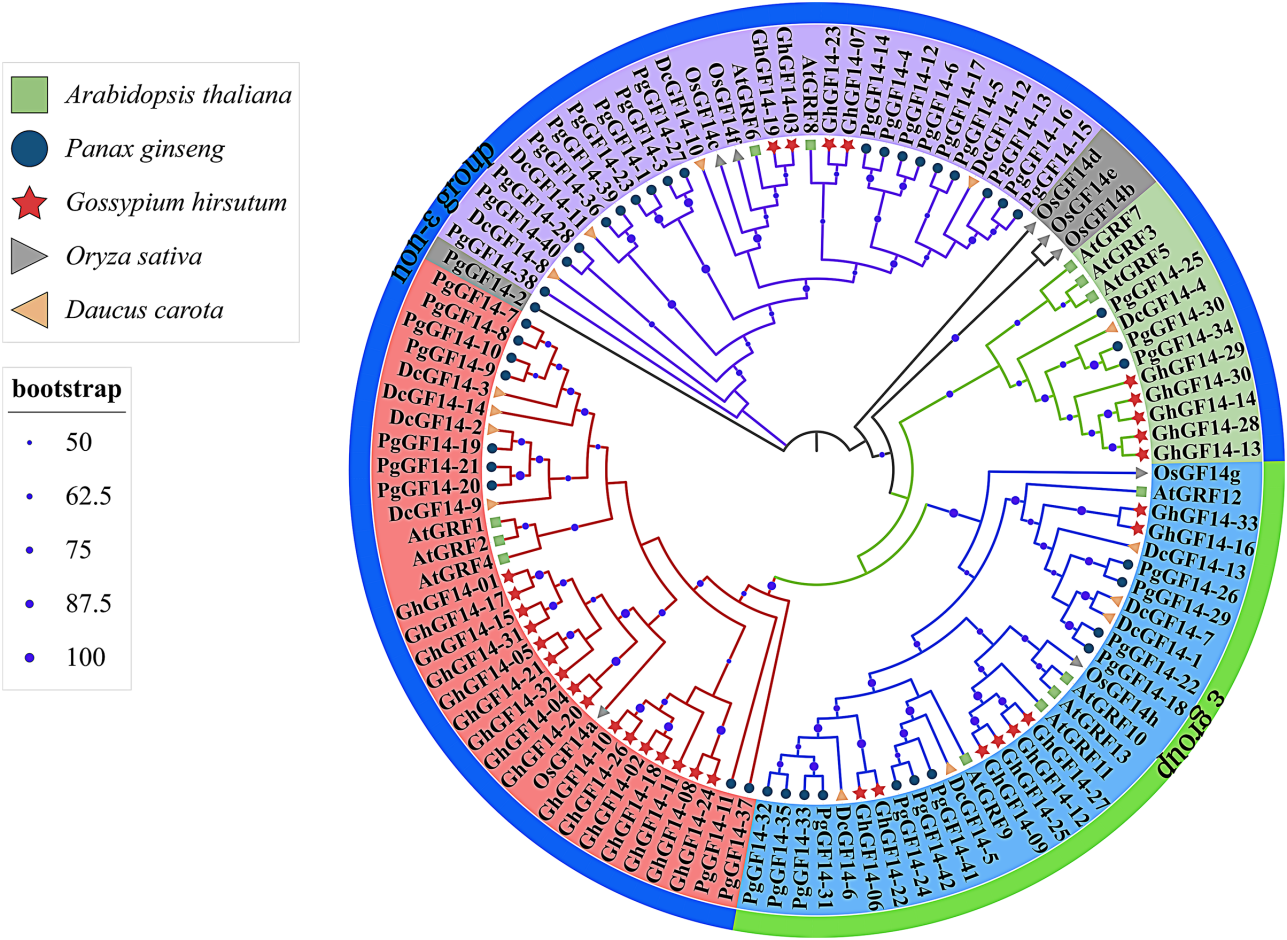
Phylogenetic tree analysis of the *14-3-3* genes in *D. carota, A. thaliana, G. hirsutum, O. sativa* and *P. ginseng*. Different colours mainly reflect multiple evolutionary branches, shape annotations represent different species, the outer circle was annotated for gene classification, solid circles located in branches represented bootstrap values, and their sizes were related to bootstrap high or low. The bootstrap display range was ≥50%.

Fourteen *D. carota 14-3-3* genes were identified in the Uniprot database and named *DcGF14-1* to *14*. The *14-3-3* genes of *A. thaliana* (*AtGRF1*–*13*) (DeLille, Sehnke & Ferl, 2001), *G. hirsutum* (*GhGF14-1*–*33*), *O. sativa* (*OsGF14a*–*g*) (Yao et al., 2007), *D. carota* (*DcGF14-1*–*14*), and *P. ginseng* (*PgGF14-1*–*42*) were used to construct a phylogenetic tree with a total of 110 genes (Fig. 4). The gene numbers and corresponding protein sequences are listed in Table S5. Except for *PgGF14-2, OsGF14b, OsGF14d*, and *OsGF14e*,106 genes were divided into four major branches, and *PgGF14s* were distributed in all branches. The phylogenetic tree indicates that most *14-3-3* genes of the selected species belong to the non-ε group, with 79 genes, including 31 of *P. ginseng*, 25 of *G. hirsutum*, nine of *D. carota*, eight of *A. thaliana*, and six of *O. sativa*. Gene homology analysis indicated that 34 of 42 *PgGF14s* existed in pairs with higher sequence similarity (Fig. S6), possibly because gene replication events produced paralogous genes. In the evolutionary tree, *P. ginseng* and *D. carota 14-3-3* genes had the highest homology, indicating they might be orthologous genes with a close genetic relationship.

### Interaction protein and transcription factor regulatory network analysis

**Figure 5:**
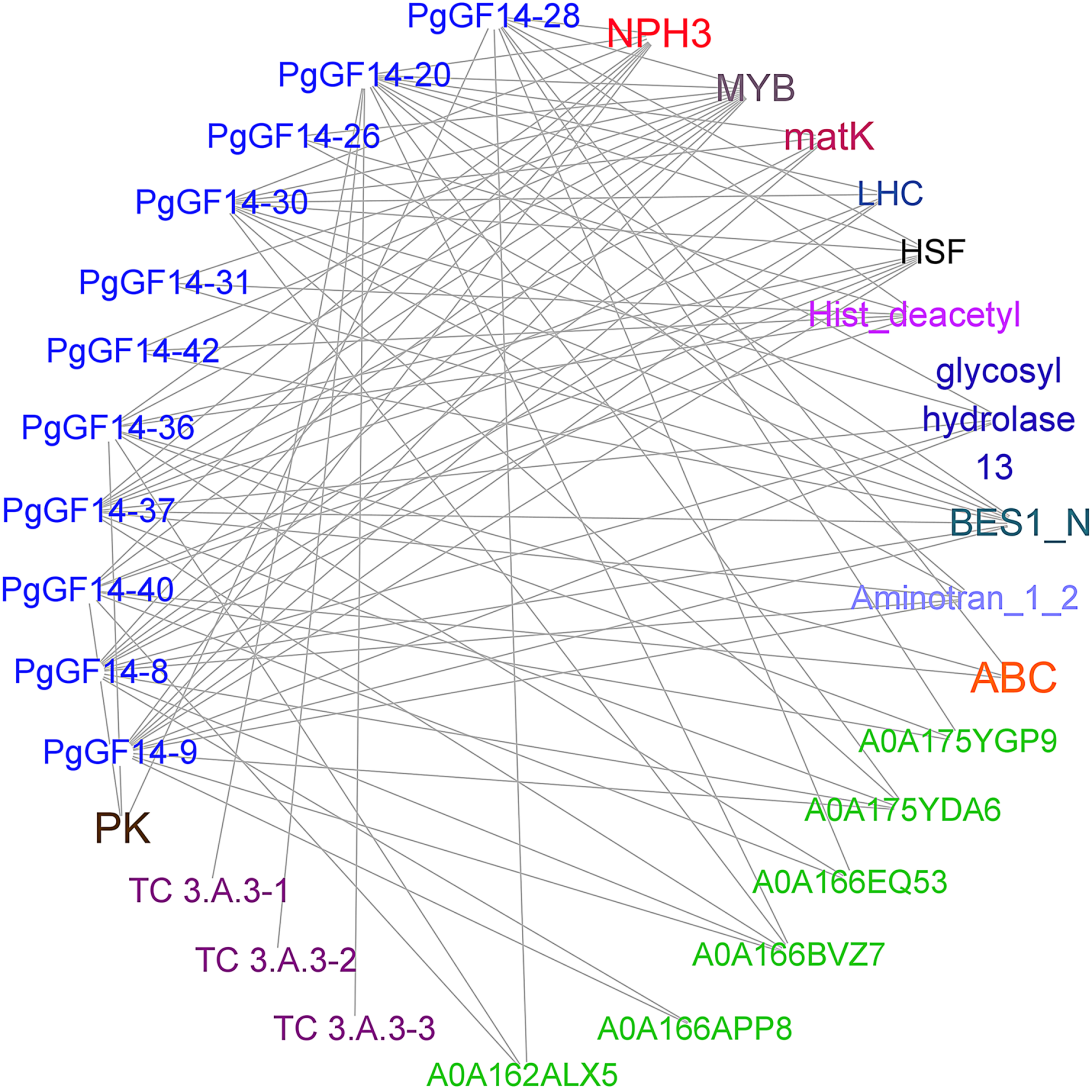
Prediction of proteins with potential interactions to *PgGF14s*. Several colours represent different gene types, while the green ones were unannotated genes in the database, and the linkage between genes indicates the potential interactions between proteins.

Sequence alignment revealed that the highest homology with the target gene in the string database was *D. carota*. The *PgGF14s* were mapped to eleven *14-3-3* proteins of *D. carota*, and some gene pairs displayed more than 90% homology (Table S6). The highest homology correspondence was used to construct an interaction network map of ginseng *14-3-3* proteins (Fig. 5, Table S6). Each node represents a different protein, and the linkage between the nodes indicates the interaction between two proteins. The results indicated that the *PgGF14s* might interact with functional genes, such as pyruvate kinase (*PK*), ATP-binding cassette transporter (*ABC*), glycoside hydrolase family 13, *LHC*, *matK*, *NPH3*, cation transport ATPase (P-type) (TC 3.A.3), and members of transcription factors *MYB*, heat shock factor (*HSF*), and Basic/Helix-Loop-Helix (*bHLH*). Some genes were predicted to interact with proteins containing gene-specific structural domains, such as the Hist_deacetyl, Aminotran_1_2, and BES1_N. The interactions between proteins were diverse. One gene may interact with multiple proteins, consistent with the functional diversity of the *14-3-3* genes and the possibility of many regulatory factors determining the gene expression. Moreover, several *14-3-3* genes were predicted to interact with other genes, such as *HSF*, *MYB*, and *NPH3*. Only *PgGF14-20* had potential interactions with all three members of the TC 3.A.3 family. The gene families and functions of some proteins that might interact with *PgGF14s* in the database remain unclear.

**Figure 6:**
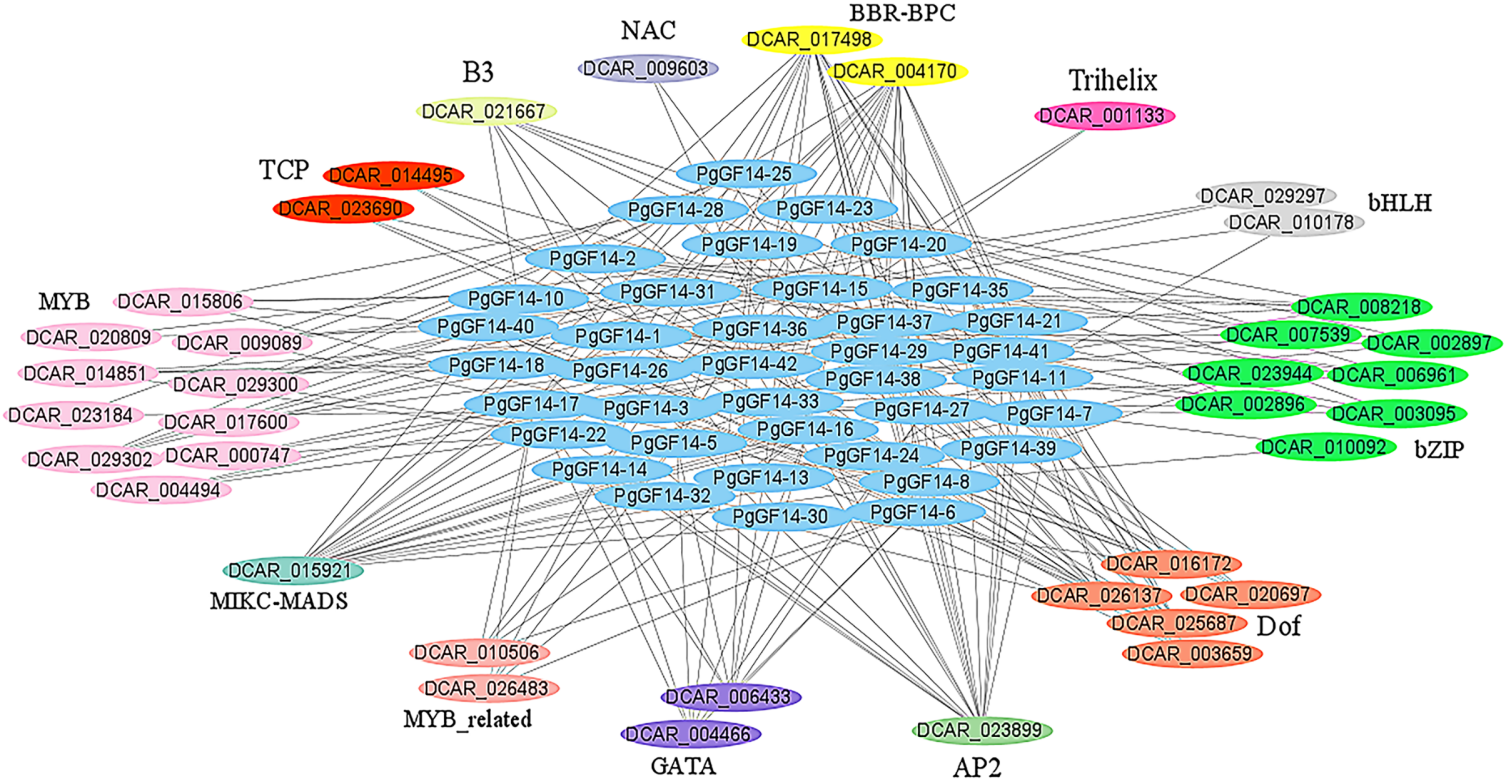
Transcription factor regulatory network analysis for *PgGF14s*. Various colours represent different genes, and the linkages between genes indicate potential regulatory relationships, and members of genes annotated as the same family were placed together.

The *PgGF14s* 600 bp promoter sequences were uploaded to the PlantRegMap database, the reference species was *D. carota*, and the other parameters were left as default. The analysis identified 355 regulations between 121 TFs and 42 ginseng *14-3-3* genes, and 38 TFs were over-represented with *p* ≤ 0.05 (Table S7). The potential regulatory relationships between 38 TFs and 42 *PgGF14s* were constructed as a network diagram, and TFs of the same type were categorized (Fig. 6). Thirteen categories of transcription factors with potential regulatory effects on *PgGF14s* were predicted, among which *MYB*, *bZIP*, and *Dof* genes accounted for a large proportion, all of which are crucial regulatory transcription factors in plants and are involved in plant abiotic stress response processes. In addition, AP2, MIKC-MADS, and BBR-BPC transcription factors may have potential regulatory roles on several *PgGF14s*.

### Transcriptome data analysis of different ginseng tissues

**Figure 7:**
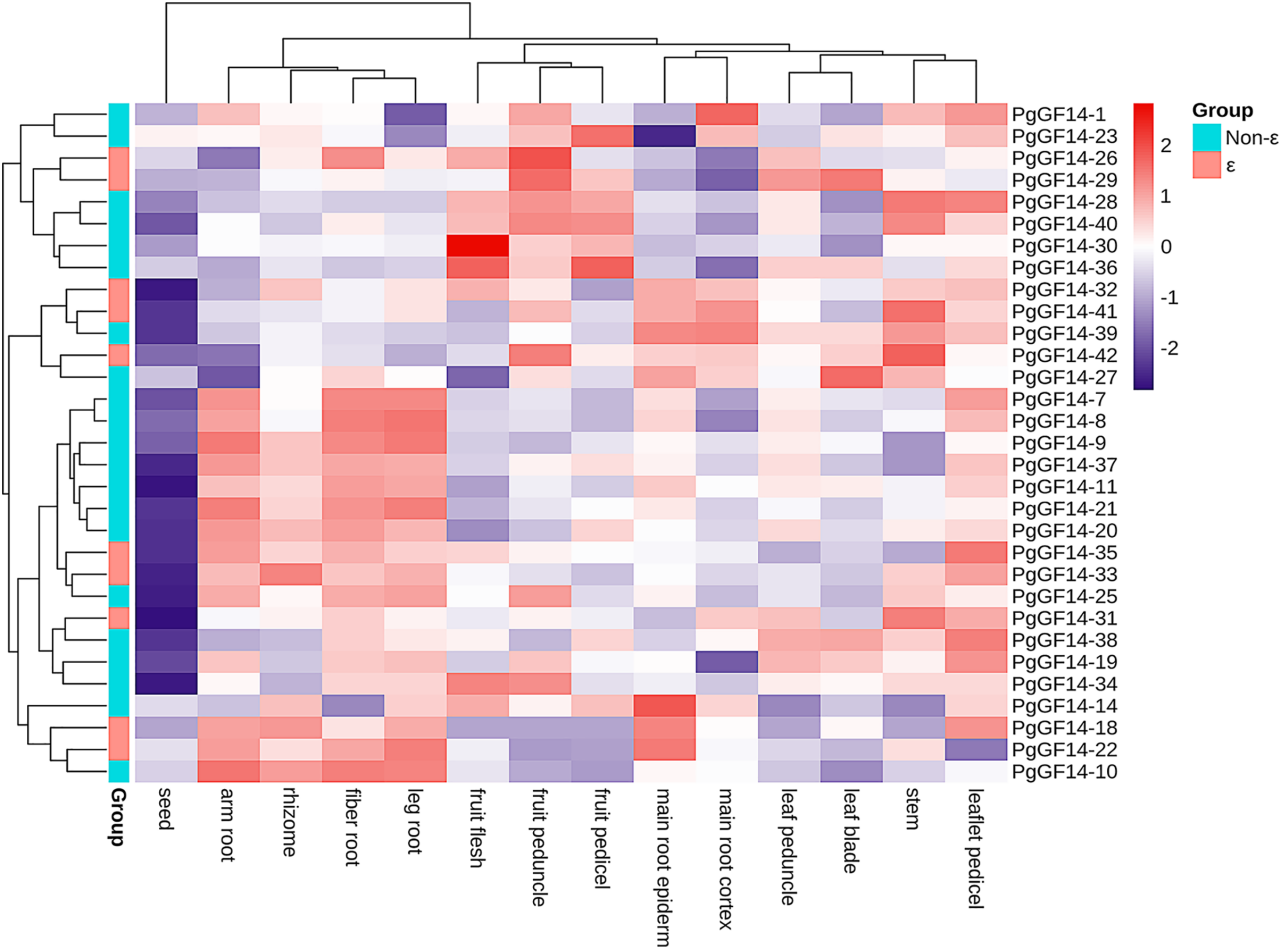
Transcriptome analysis of *PgGF14s* expression in different tissues of ginseng. Gene expression levels were reflected as normalized TPM values in Log_2_ (TPM+1), clustered based on the euclidean method and reflected in different colours.

The species used for transcriptome analysis was a four-year-old Jilin ginseng ‘Damaya’, whose following parts were analyzed: fiber root (SRR2952867), leg root (SRR2952868), main root epiderm (SRR2952869), main root cortex (SRR2952870), rhizome (SRR2952871), arm root (SRR2952872), stem (SRR2952873), leaf peduncle (SRR2952874), leaflet pedicel (SRR2952875), leaf blade (SRR2952876), fruit peduncle (SRR2952877), fruit pedicel (SRR2952878), fruit flesh (SRR2952879), and seed (SRR2952880). The raw TPM values of *PgGF14s* in different tissue sites are displayed in Table S8. A heat map for gene expression was drawn based on the TPM of *PgGF14s* in log_2_ (TPM+1) (Fig. 7). Nine genes were not detected in this sequencing (*PgGF14-2, 4, 5, 6, 12, 13, 15, 17*, and *24*). *PgGF14-3* and *PgGF14-16* were expressed just in one tissue and with very low gene abundance, so these genes were not represented in the heat map. Among the remaining 31 genes, 26 (83.9%) were expressed in all tissues, and five (16.1%) were expressed in most tissues. The gene abundance of different members varied greatly. For example, *PgGF14-25*, *30*, and *34* were highly expressed, *PgGF14-20*, *33*, and *37* were moderately expressed, and *PgGF14-22*, *23*, and *36* were lowly expressed. Overall, the expression levels of *PgGF14s* in different organs were variable. The *14-3-3* gene abundance was low in seeds but relatively high in roots, stems, leaves, and fruits. Meanwhile, the expression of *14-3-3* genes displayed tissue specificity. *PgGF14-30* displayed a high abundance in different tissues, especially in the fruit flesh. Some genes were expressed at relatively high levels in the arm root, fiber root, leg root, and rhizome, such as *PgGF14-7*, *8*, *9*, *11*, and *20*. *PgGF14-28*, *30*, and *36* were more abundant in the fruit flesh, fruit peduncle, and fruit pedicel.

### Expression analysis of *14-3-3* genes under heat stress

**Figure 8:**
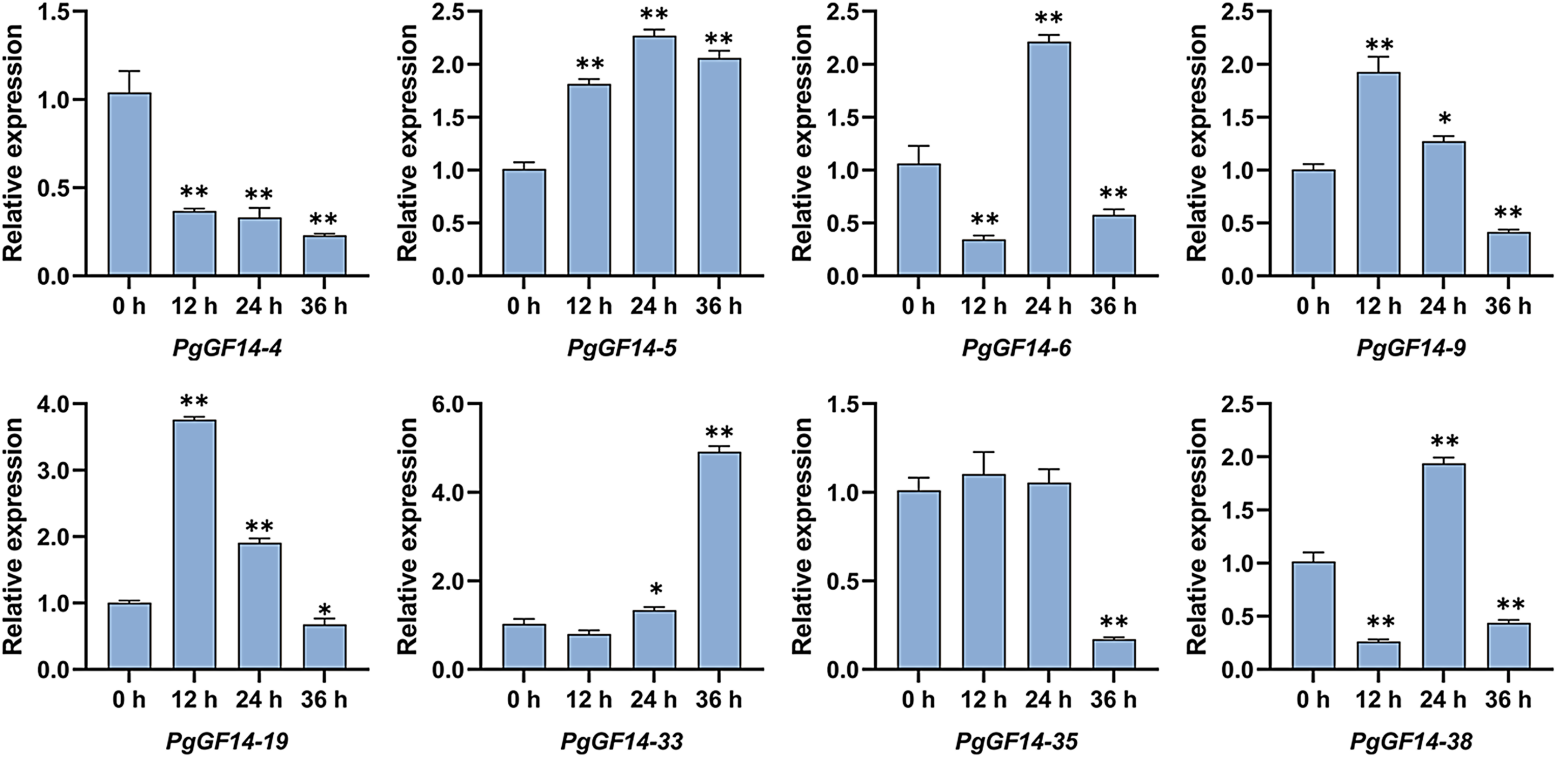
Typical expression patterns of *PgGF14s* under heat stress with qRT-PCR. The expression levels of *PgGF14s* in ginseng histoculture seedlings were measured by qRT-PCR at 0, 12, 24 and 36 h under heat stress. The asterisk above the bar chart represents the statistically significant differences between the stress groups and the control (*p < 0.05, **p < 0.01). Error bars represent the SD values of biologic replicates.

**Figure 9:**
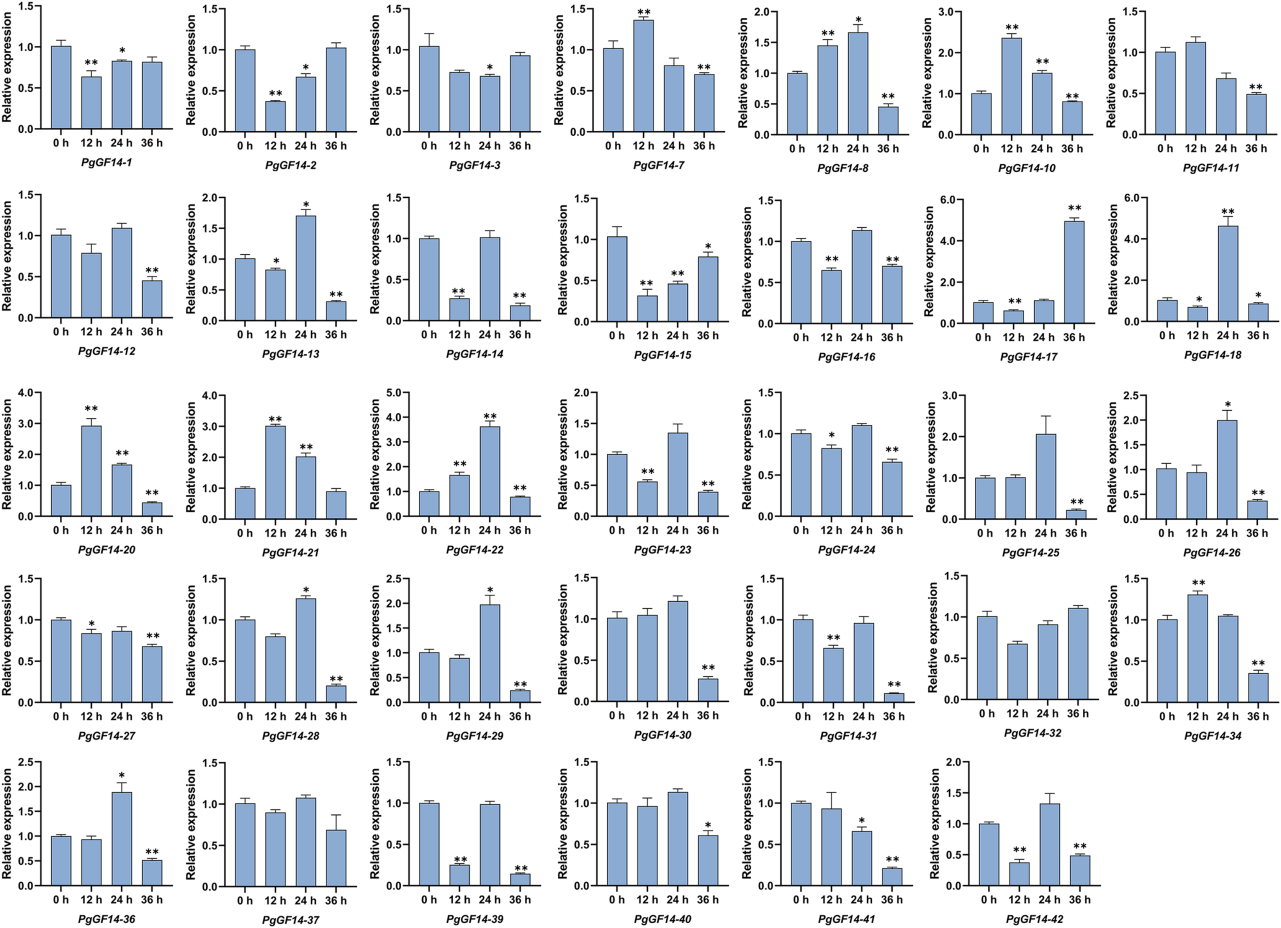
The qRT-PCR results of the remaining *PgGF14s*. The asterisk above the bar chart represents the statistically significant differences between the stress groups and the control (*p < 0.05, **p < 0.01). Error bars represent the SD values of biologic replicates.

All *14-3-3* genes were detected in the experiment, and the melting curves indicated specificity to the primers and genes. Raw data for qRT-PCR and specific *p*-values are displayed in Table S9. The results of the qRT-PCR analysis indicated that, overall, the expression patterns and levels of *PgGF14s* differed between the three treatment times at 35 °C. Representative examples of *PgGF14s* with different expression patterns are displayed in Fig. 8, and the relative expression for the remaining genes are displayed in Fig. 9. Several genes—*PgGF14-6*, *13*, *14*, *16*, *18*, *23*, *24*, *31*, *38*, *39* and *42*—were downregulated at 12 h of treatment; their expression rebounded at 24 h and decreased again at 36 h. The expression levels of *PgGF14-7*, *8*, *9*, *10*, *19*, *20*, *21*, and *34* were high at 12 and 24 h and lower at 36 h, displaying a trend of rising first and falling later. Some genes responded significantly to heat at a particular time point. *PgGF14-19* was significantly upregulated at 12 h of high-temperature stress, with a 3.759 times higher expression than the control. *PgGF14-18* and *22* were remarkably upregulated at 24 h of stress, with 4.635 and 3.629 times higher expression than the control, respectively. The expression levels of *PgGF14-12*, *25*, *26*, *28*, *29*, *30*, *35*, and *36* were not very affected at 12 and 24 h treatment times but were significantly downregulated at 36 h of stress. In addition, *PgGF14-4* was significantly downregulated at different treatment times, and its expression tended to decrease with increased stress time. On the other hand, *PgGF14-5* was significantly upregulated at different treatment times. The relative expression of *PgGF14-4* and *PgGF14-5* also changed considerably at different processing times. The same trend and apparent response at several time points suggest that *PgGF14-4* and *PgGF14-5* may be continuously involved in the response of ginseng to high-temperature stress and may have a more critical regulatory role. Among the 42 *14-3-3* genes in ginseng, 38 responded more significantly to the high-temperature stress, indicating that the ginseng *14-3-3* gene family may be involved in response to abiotic stresses, particularly heat stress. The differences in expression patterns and levels of different members also indicated the diversity and variability of *PgGF14s* functions.

## Discussion

Ginseng is a valuable herbal resource. However, its growth and development are greatly limited by region and climate, making it more susceptible to various environmental factors. Ginseng is extraordinarily cold-tolerant but extremely intolerant to high temperatures. Under heat stress, photosynthesis, cell structure or metabolism, and signal transduction are severely impacted. Since plants will actively carry out self-protection and regulation to resist the adverse external environment at the genetic level, exploring the functional genes involved in response to high-temperature stress in ginseng is crucial.

Given the vital role of the *14-3-3* gene family in abiotic stress responses, 42 ginseng *14-3-3* genes were identified based on the joint action of HMM model, SMART, BLASTp, and NCBI-CDD and were renamed *PgGF14-1* to *PgGF14-42*, all containing conserved structural domains of the *14-3-3* family (Fig. S1). Protein sequence alignment indicated that the sequences of *PgGF14s* were highly conserved, except for the C-terminus part of the protein that displayed high variability (Fig. S4), similar to previous studies (Jones, Ley & Aitken, 1995). The large *14-3-3* family of ginseng had significantly more members than other identified *14-3-3* gene families, such as that in *G. max* (Wang et al., 2019), *C. sinensis* (Lyu et al., 2021), *O. sativa* (Yao et al., 2007), *M. acuminat* (Li et al., 2016), and *S. lycopersicum* (Xu & Shi, 2006). Ginseng is an ancient relict plant that has experienced 65 million years and has undergone a longer and more complex evolutionary process. A large number of the *14-3-3* gene family may be related to gene replication events and the retention of favourable mutations under selection pressure.

The structure of *14-3-3* proteins is essential for their phosphorylation and interaction with target proteins. Structural prediction of *PgGF14s* indicated that the ginseng *14-3-3* proteins mainly had an α-helix basic structure and mostly formed dimers (Figs. S2 and S3). The result was consistent with previous studies on the structure of *14-3-3* proteins. *14-3-3* proteins have multiple subtypes with different functions and are effective as dimers (Messaritou, Grammenoudi & Skoulakis, 2010). Most monomers comprise nine reverse parallel α-helix and are connected by a loop structure to form C- and N-terminal structures (Yang et al., 2006). The C-terminal domain is directly involved in the protein-protein interaction, impacting the protein conformation and its binding with the target protein. Meanwhile, the prominent N-terminal domain directly affects the binding between proteins and membrane structure (Manak & Ferl, 2007).

The intron-exon structure of genes is essential in clarifying the species’ evolutionary relationships (Xu et al., 2019). Based on this structure, ginseng *14-3-3* genes were classified into ε and non-ε classes within 11 in ε and 31 in non-ε (Figs. 1 and 4). Generally, *14-3-3* genes within the same branch had a brilliant structure consistency of their motif, indicating a structural and evolutionary conservation of this gene family (Fig. 1). However, the exon numbers of *PgGF14-41* and *42* were 16 and 17, respectively, and the outcome differed from the conventional structure of the *14-3-3* genes. The variance in gene structure might generate from duplication events during evolution or insertion/deletion of introns and exons under selection pressure (Yu et al., 2022).

The phylogenetic tree lays a foundation for functional prediction and genetic analysis for gene families. In the evolutionary analysis of ginseng (Fig. 4), the ε-class genes of *G. hirsutum*, *A. thaliana*, *O. sativa*, and *D. carota* were clustered into one class. Members of the ε-class were consistent with previous studies (DeLille, Sehnke & Ferl, 2001; Yao et al., 2007). Both *P.ginseng* and *D. carota* belong to the Umbellales family. Studies have demonstrated that *D. carota* is closely related to ginseng based on assembled reference genomes (Wang et al., 2022). In the evolutionary relationship of the target gene, ginseng and *D. carota* with close genetic relationships embodied high homology in *14-3-3* genes. Compared with the other three species, *PgGF14s* had a distant clustering relationship with the *O. sativa 14-3-3* genes, probably because *P. ginseng*, *A. thaliana*, and *G. hirsutum* are dicotyledons, and *O. sativa* a monocotyledon, and the evolution and development of genes differ between monocots and dicots.

Plant gene expression results from the interaction between multiple trans-factors and *cis*-acting elements. The predictive analysis of *cis*-acting elements lays a theoretical foundation for researching gene function and exploring potential mechanisms. Analysis of the *cis*-acting elements for *PgGF14s* found many hormone and stress response elements and regulatory elements upstream of the gene, indicating that *PgGF14s* were involved in various biological processes and played an essential role in plant responses to abiotic stress (Fig. 2). The results of GO annotation and enrichment also confirmed that observation (Fig. 3).

The expression patterns of *PgGF14s* in different tissues indicated that ginseng *14-3-3* were expressed in different tissues and organs. However, the differences between tissues and organs may indicate that *PgGF14s* are involved in different life activities and development regulations. The expression abundance of *PgGF14s* was high in roots, stems, leaves, and fruits but low in seeds. Some genes were highly expressed in roots and rhizomes, such as *PgGF14-7*, *8*, *9*, *11*, and *20*. *PgGF14-28*, *30*, and *36* were mainly highly expressed in fruits (Fig. 7). *PgGF14s* might participate in the development of underground parts and fruit maturation in ginseng. Expression patterns and functions of *14-3-3* genes appear to differ between species. *14-3-3* genes in *B.distachyon* were mainly expressed in roots and leaves. In *C. sinensis*, *CitGF14a*, *c*, *d*, *h*, and *i* were highly expressed in roots, and *CitGF14c*, *e*, and *h* were highly expressed in leaves. The high expression of *CitGF14d* in flowers and roots implied the importance of *14-3-3* in flower and root development. The 16 detected *SGF14s* were highly expressed in embryo tissues, indicating that *SGF14s* played an essential role in seed growth and development (Li & Dhaubhadel, 2011). In another report on *G. max 14-3-3s*, the expression level of *GmGF14s* in vegetative organs was generally higher than in reproductive organs. *GmGF14k*, *GmGF14p*, and *GmGF14o* were highly expressed in the root, pod, and stem, respectively (Wang et al., 2019). *OsGF14c* was uniformly expressed in all tissues in rice, *OsGF14b* was abundant in the root, and *OsGF14d* and *OsGF14g* were highly expressed in mature leaves and stems. *14-3-3* genes of *M. indica* were expressed in all tissues, and some genes were expressed explicitly in buds, flowers, and young leaves (Xia et al., 2022). However, whether there is variability in the expression patterns of target genes in different varieties and developmental stages needs to be further investigated.

Currently, the information on ginseng species is not included in the String and PlantRegMap databases. Therefore, *PgGF14s* were mapped to the carrot’s high homology *14-3-3* genes to predict the interaction proteins and regulatory network. Under a high interaction score, 20 genes that may interact with *PgGF14s* were screened (Fig. 5). Some genes have been verified to interact with *14-3-3* or participate in abiotic stresses, such as *MYB* and *HSF*. *HSF* transcription factors are crucial in the plant heat stress response. Studies in *A. thaliana* indicated that *HSFA1* could activate a series of target genes, including many heat shock proteins under heat stress (Anckar & Sistonen, 2011). The interaction between *G. max 14-3-3* proteins and *MYB* transcription factors can regulate the nuclear localization of *GmMYB176*, thereby affecting the biosynthesis of isoflavones (Li, Chen & Dhaubhadel, 2012).

The regulation of transcription factors has a direct effect on gene expression. The regulatory network prediction indicated that *PgGF14s* might be regulated by various transcription factors, such as *MYB*, *bZIP*, *bHLH*, *Dof*, and *GATA* (Fig. 6). Those transcription factors are engaged in regulating the response to abiotic stresses in plants. VIGS assay and protein-protein interaction analysis confirmed that *TaGRF6-A* of wheat interacted with *MYB* transcription factors (*TaMYB64*) and facilitated the salinity tolerance of plants (Shao et al., 2021). The *GATA* transcription factor had a significant differential expression in response to high-temperature stress in *Brassica juncea* (Bhardwaj et al., 2015). Through macroarray (dot blot) analysis, the expression of the *Dof* gene in *Carthamus tinctorius* L. increased significantly under drought stress (Thippeswamy et al., 2013). The *bZIP* genes were confirmed to be involved in responses to abiotic stresses, such as heat, low temperature, drought, and pathogen-mediated biotic stress (Jakoby et al., 2002; Sornaraj et al., 2016). The *O. sativa bHLH* gene can respond to drought stress (Li et al., 2006), and the overexpression of the *OsbHLH148* gene increased the resistance of *O. sativa* to drought (Seo et al., 2011). The potential interactions between *14-3-3s* and these transcription factors imply that these factors may regulate the response of the *PgGF14s* to stress. By analyzing the regulatory network of *PgGF14s* transcription factors, we can predict the potential reciprocal proteins and binding sites for target genes, which lay the theoretical foundation and research direction for ginseng protein interactions.

The qRT-PCR analysis of *PgGF14s* under high-temperature indicated that about 90% of *PgGF14s* responded to heat stress. The expression of *PgGF14s* under high-temperature conditions reflected a variety of patterns and mainly included the following patterns: down-up-down, up-down-up, up-down, and down-up regulations. Some genes strongly respond to high temperatures at a particular time (Figs. 8 and 9). Existing research indicates that heat stress causes differential expression of the *14-3-3* genes.

In *B. distachyon*, most *BdGF14s* were significantly upregulated under high temperature, and the expression levels of *BdGF14a*, *b*, *d*, and *e* in roots and leaves increased strongly under heat stress (Cao et al., 2016). The expression levels of *OsGF14b*, *c*, and *d* were higher under a 42 °C stress (Yao et al., 2007). Under heat stress, when photosynthesis is inhibited by 20–30%, the expression of *PtGRF11a* in *Populus trichocarpa* is upregulated (Tian et al., 2015). Among the 11 *Vitis vinifera 14-3-3* genes, six *VviGRFs* had an apparent response to heat stress (Cheng et al., 2018). All genes of the *14-3-3* family in wheat were downregulated under heat stress, whether for one or six hours (Guo et al., 2018). These results provide evidence for the potential role of *14-3-3* genes in regulating plant stress, including high temperature.

Currently, the response mechanism of *14-3-3* genes to high temperature is unclear. The change in *14-3-3* expression may activate or inhibit some metabolic pathways or enzyme activities to prevent high-temperature damage to plants. Meanwhile, the response of *14-3-3* to heat stress may be related to their involvement in ABA-dependent, calcium-dependent protein kinase (CDPK) and other critical signaling pathways. Multiple expression patterns exhibited by *PgGF14s* under heat stress also implied the diversity of functions and mechanisms in ginseng, while *PgGF14-4* and *PgGF14-5* may be the more critical response genes. Moreover, proteins and transcription factors potentially interacting with *PgGF14s* were predicted, some of which have been confirmed to interact with *14-3-3* proteins or play an essential role in plant response to abiotic stress. But in this research, heat shock response (HSE) elements were not predicted in the upstream regulatory region of *PgGF14s* that respond to high temperature including *PgGF14-4* and *PgGF14-5*, it is speculated their reaction to heat stress may not be a direct effect. The *PgGF14s* might indirectly respond to high-temperature stress by regulating, interacting and binding with other proteins or elements such as HSE.

## Conclusions

In this study, 42 *PgGF14s* (*PgGF14-1*–*PgGF14-42*) were identified in the ginseng genome, and the classification and evolutionary relationship of *PgGF14s* were determined based on intron-exon structure, motif, and gene evolution analysis. Bioinformatics and multiple database analyses were conducted to predict the physicochemical properties and structures of the proteins. Analysis of *cis*-acting elements, GO enrichment, interaction protein, and transcription factor regulatory networks indicated that *PgGF14s* participate in processes that respond to adversity stress and regulate growth and development, material metabolism, and signal transduction. The protein-protein interaction and transcription factor network analysis laid a theoretical foundation for studying PgGF14 protein function and mechanism. RNA-seq analyses of different tissue sites indicated that *PgGF14s* were expressed in the whole plant but differed in abundance. The qRT-PCR analysis further indicated that most *PgGF14s* responded to high-temperature stress. From the perspective of gene expression patterns, *PgGF14-4* and *PgGF14-5* may be the more critical genes in the *14-3-3* family in response to heat stress. Combining the results of qRT-PCR with the analysis of promoter elements and reciprocal proteins for *PgGF14s*, it is speculated that the response of genes to high temperature stress may be indirect. The above results indicate that *PgGF14s* are involved in various plant activities and have potential regulatory effects in resisting adverse environmental factors, including high temperatures. In conclusion, this study provides methods for functional gene research and lays a foundation for deeper exploration of the *PgGF14s* in abiotic stress contexts in ginseng.

## Supplemental Information

10.7717/peerj.15331/supp-1 Supplemental Information 1 List of qRT-PCR primers for *PgGF14s*.

10.7717/peerj.15331/supp-2 Supplemental Information 2 Physicochemical properties and subcellular localization of *PgGF14s*.

10.7717/peerj.15331/supp-3 Supplemental Information 3 Details of the types and functions to *PgGF14s* cis-acting elements. The locations of upstream promoter elements of *PgGF14s* and outlines their primary functions and the motifs they contain.

10.7717/peerj.15331/supp-4 Supplemental Information 4 GO annotation and GO enrichment information of *PgGF14s*. All GO annotations for *PgGF14s* by the eggNOG and the filtering results at level 2.

10.7717/peerj.15331/supp-5 Supplemental Information 5 Multiple species*14-3-3* protein FASTA sequences. The protein sequences of all genes used to construct the evolutionary tree.

10.7717/peerj.15331/supp-6 Supplemental Information 6 Gene correspondence and information on interacting proteins. Homology of *P. ginseng* and *D*.*carota* 14-3-3 genes, functional annotation of target proteins and proteins with potential interactions with *PgGF14s* are listed in this table.

10.7717/peerj.15331/supp-7 Supplemental Information 7 Prediction of *PgGF14s* transcription factor regulation and construction of an interoperability network. Interaction relationships in the table were all the predicted interactions. The over-represented TFs were the main interactions after enrichment and combined with transcription factor annotation to plot the interactions network.

10.7717/peerj.15331/supp-8 Supplemental Information 8 Raw data of *PgGF14 s*expression in different tissues. The expression of *PgGF14s* in different tissues of ginseng were measured as homogenized TPM values, and the Log_2_ (TPM+1) values used for heat map construction were listed.

10.7717/peerj.15331/supp-9 Supplemental Information 9 Raw data for qRT-PCR Cq values and statistical analysis. The raw Cq value data for qRT-PCR with the statistically significant differences and SD of three biological replicates and two technical replicates.

10.7717/peerj.15331/supp-10 Supplemental Information 10 Conserved *14-3-3* structural domain of *PgGF14s*. The figure showed the protein-conserved structural domains of *PgGF14s* and their location information in protein sequences, all genes identified contained the*14-3-3* structural domain.

10.7717/peerj.15331/supp-11 Supplemental Information 11 Secondary structure of *PgGF14s*. The images provided information on the secondary structures of *PgGF14-1* to *PgGF14-42* in order, including the percentage of each secondary structure in the gene.

10.7717/peerj.15331/supp-12 Supplemental Information 12 Tertiary structure of *PgGF14s*. Protein sequences were uploaded to the Swiss-Model database and matched to the most consistent protein structures, which were listed in order.

10.7717/peerj.15331/supp-13 Supplemental Information 13 Multiple sequence alignment of *PgGF14s* proteins in ginseng. The proteins of *PgGF14s* were subjected to multiple sequence alignments and trimmed for gaps. The size and type of amino acid symbols above the sequence reflected the degree of sequence conservation.

10.7717/peerj.15331/supp-14 Supplemental Information 14 Sequence logo of motifs in *PgGF14s*. Motif 1 to Motif 10 reflected the characteristic sequence in *PgGF14s*.

10.7717/peerj.15331/supp-15 Supplemental Information 15 Sequence alignment of paired genes in *PgGF14s*. DNAMAN aligned the CDS of gene pairs for sequence comparison. Set highlight homology level range to ≥ 50%. Identity values indicated the gene homology.

## Additional Information and Declarations

### Data Availability

The following information was supplied regarding data availability: The fluorescence quantification, statistical analysis and other relevant raw data are available in the Supplemental Files.
