# Supplementary figures and images for: Molecular analysis of the 14-3-3 genes in Panax ginseng and their responses to heat stress
Source: PeerJ. 2023 May 9;11:e15331. doi: 10.7717/peerj.15331 (PMC10178371; doi:10.7717/peerj.15331)

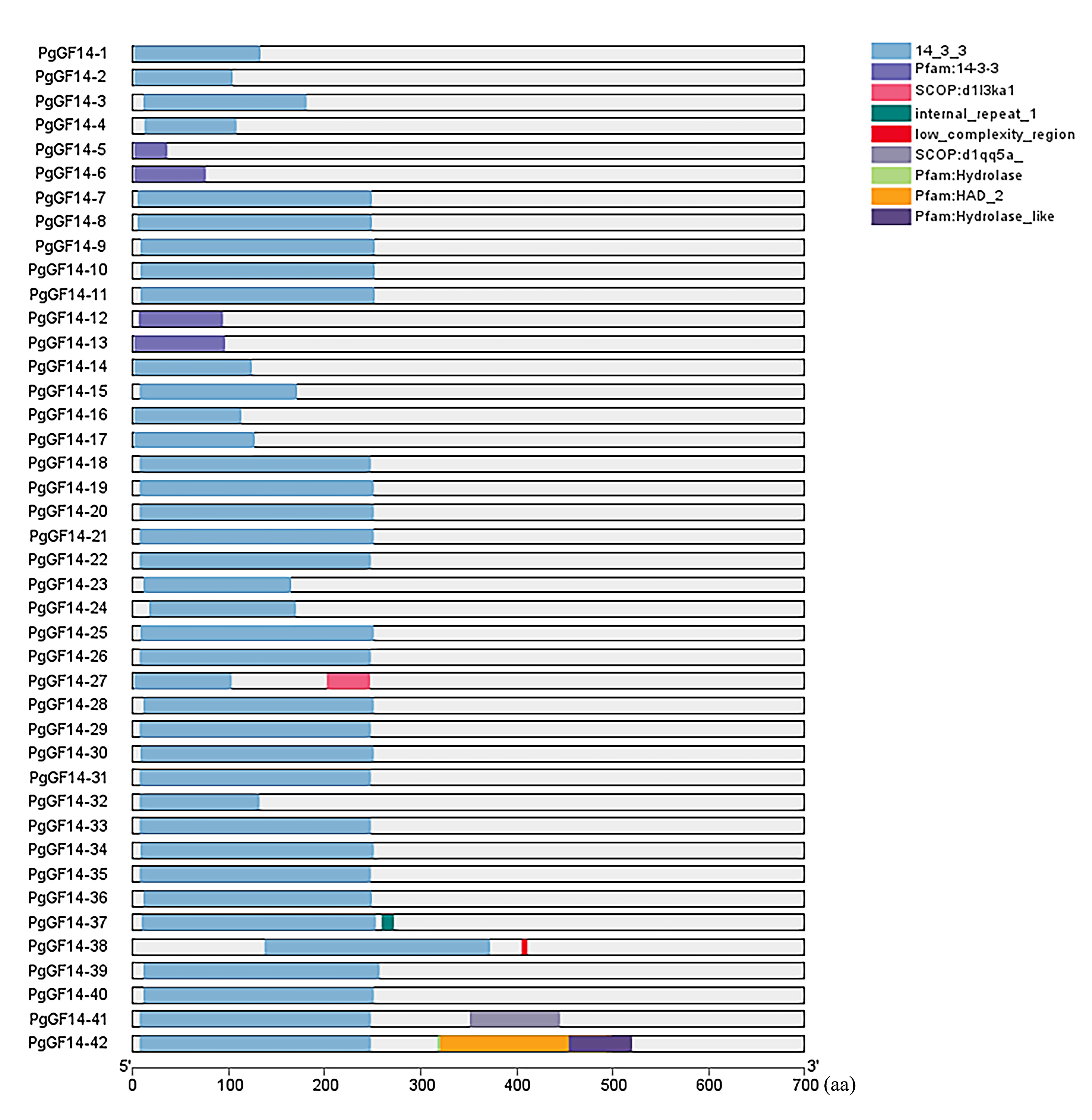

Supplement: Supplemental Information 10 — The figure showed the protein-conserved structural domains of PgGF14s and their location information in protein sequences, all genes identified contained the14-3-3 structural domain. [file peerj-11-15331-s010.png]

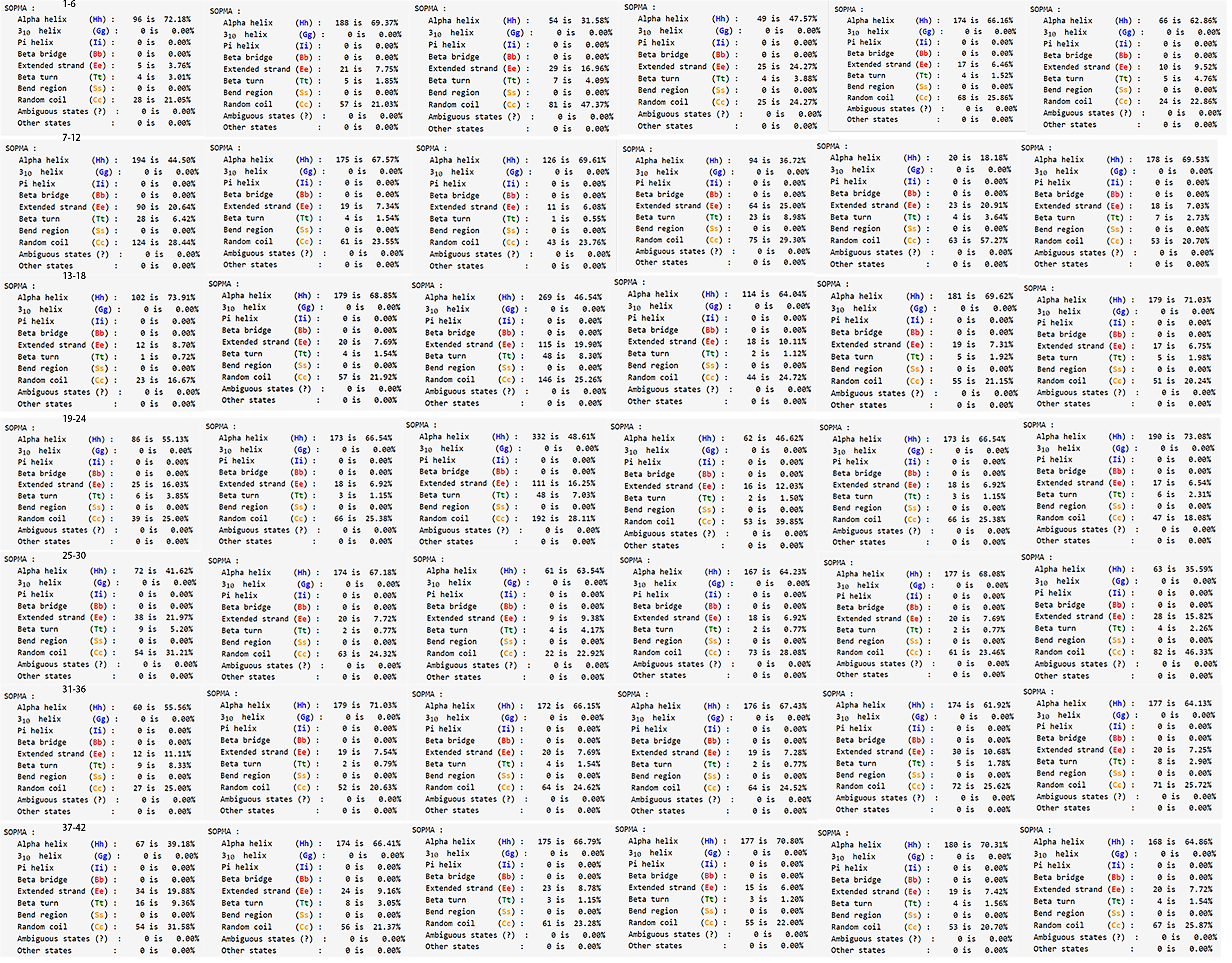

Supplement: Supplemental Information 11 — The images provided information on the secondary structures of PgGF14-1 to PgGF14-42 in order, including the percentage of each secondary structure in the gene. [file peerj-11-15331-s011.png]

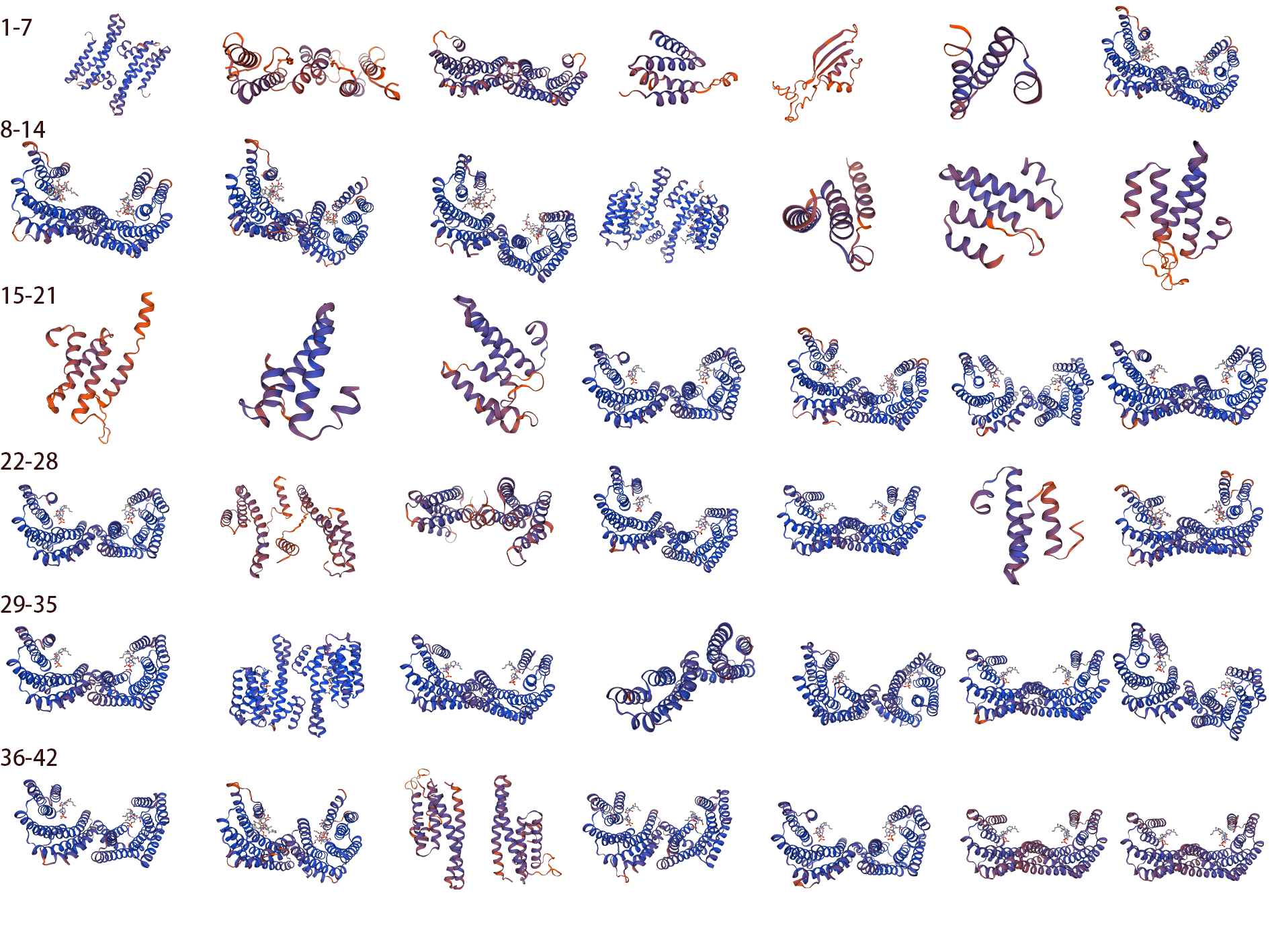

Supplement: Supplemental Information 12 — Protein sequences were uploaded to the Swiss-Model database and matched to the most consistent protein structures, which were listed in order. [file peerj-11-15331-s012.png]

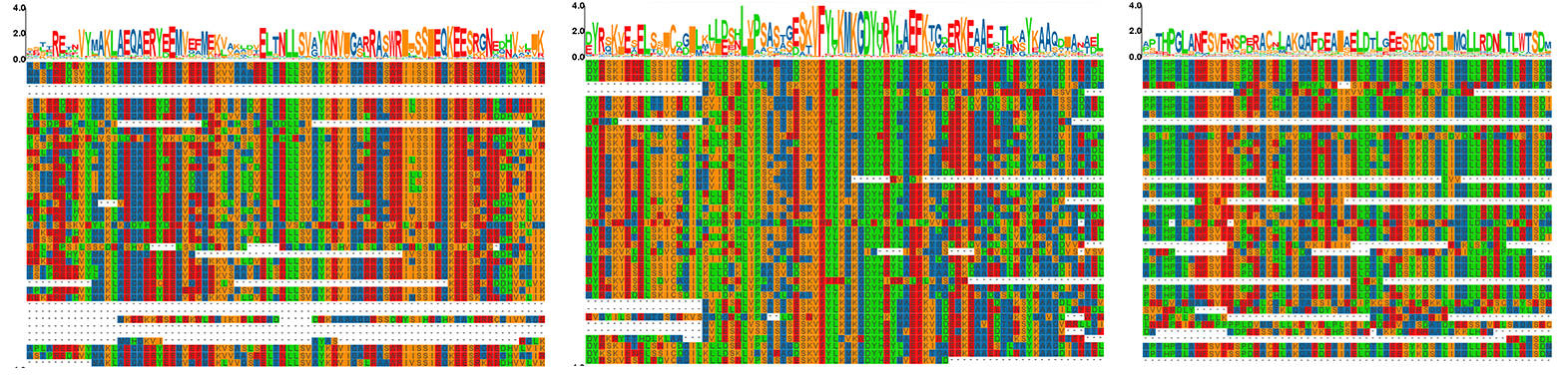

Supplement: Supplemental Information 13 — The proteins of PgGF14s were subjected to multiple sequence alignments and trimmed for gaps. The size and type of amino acid symbols above the sequence reflected the degree of sequence conservation. [file peerj-11-15331-s013.png]

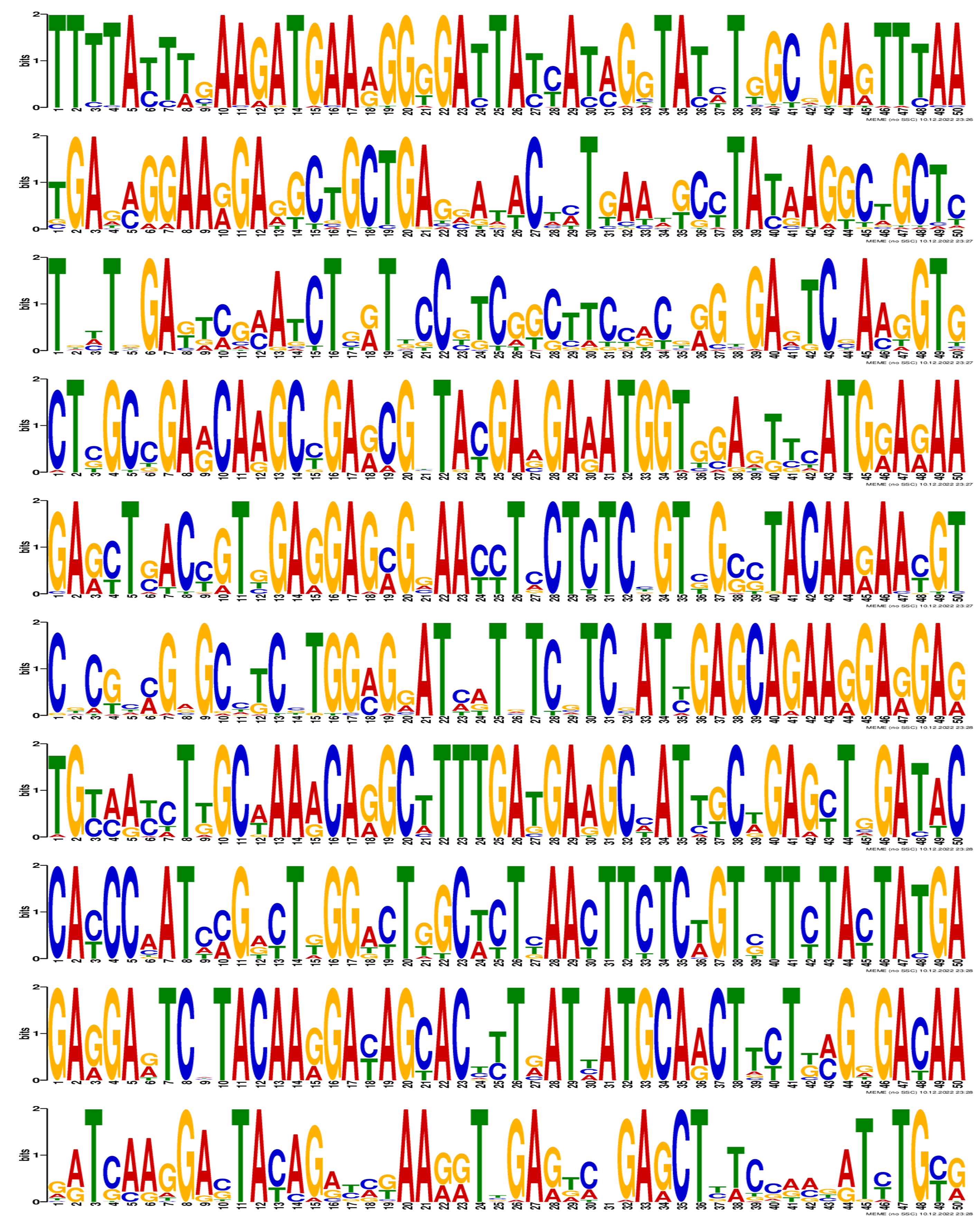

Supplement: Supplemental Information 14 — Motif 1 to Motif 10 reflected the characteristic sequence in PgGF14s. [file peerj-11-15331-s014.png]

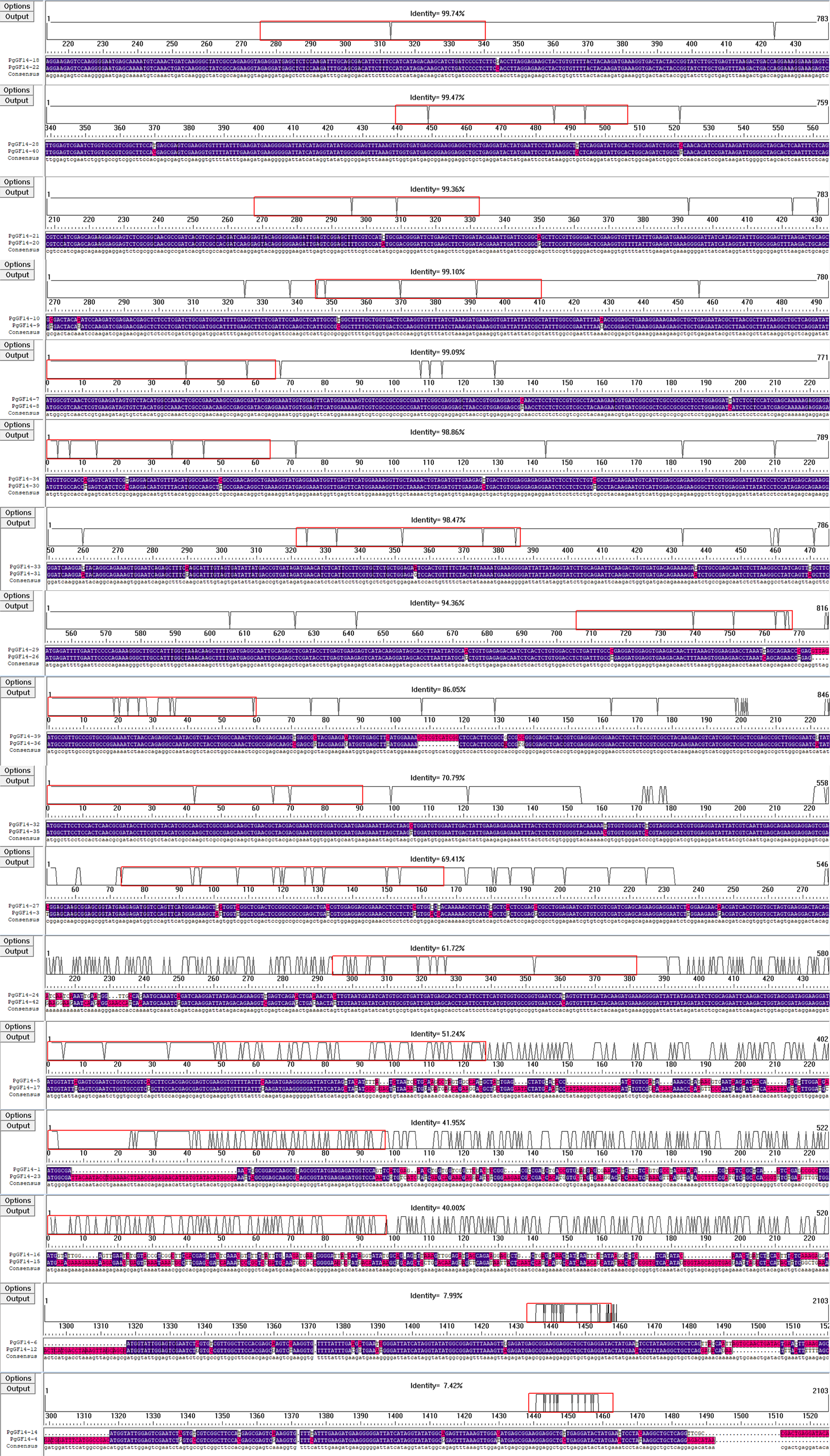

Supplement: Supplemental Information 15 — DNAMAN aligned the CDS of gene pairs for sequence comparison. Set highlight homology level range to ≥ 50%. Identity values indicated the gene homology. [file peerj-11-15331-s015.png]
